# Unveiling key genetic loci and candidate genes for brown spot disease resistance in rice based on QTL analysis

**DOI:** 10.1038/s41598-025-33890-y

**Published:** 2026-01-29

**Authors:** Dan-Dan Zhao, Hyunjung Chung, Muhammad Farooq, Nam-Gu Kim, Soo Yeon Choi, Shinhwa Kim, Sang-Min Kim, Jang-Ho Lee, Xiao-Xuan Du, Kyung–Min Kim

**Affiliations:** 1 Crop Environment Research Division, National Institute of Crop and Food Science, Rural Development Administration, Wanju, 55365 Korea; 2 College of Marine and Bioengineering, YanCheng Institute of Technology, 211 Jianjun East Road, Yangcheng City, Jiangsu Province 224051 China; 3 Coastal Agriculture Research Institute, Kyungpook National University, Daegu, 41566 Korea; 4 Department of Applied Biosciences, Kyungpook National University, Daegu, 41566 Korea

**Keywords:** Rice brown spot, Quantitative trait locus (QTL) analysis, Candidate genes, Ribosome-inactivating protein, Defense mechanisms, Marker-assisted selection, Biotechnology, Genetics, Molecular biology, Plant sciences

## Abstract

Rice brown spot, a widespread disease caused by the fungus *Bipolaris oryzae* (teleomorph: *Cochliobolus miyabeanus*), significantly diminishes both grain yield and quality. Developing resistant rice cultivars remains the most sustainable and effective strategy for managing this disease. In this study, we conducted quantitative trait locus (QTL) analysis to identify genetic loci and candidate genes associated with brown spot resistance. A mapping population was generated from a cross between the resistant variety ‘Cheongcheong’ and the susceptible variety ‘Nagdong’. Disease evaluations were performed following inoculation with a mixture of six *Cochliobolus miyabeanus* isolates collected in 2023 to represent current field diversity, and disease severity was assessed seven days post-inoculation using a 0–9 scale. The QTL analysis revealed a new major locus on chromosome 3 linked to brown spot resistance, between markers RM15749 and RM15689. Within this genomic interval, 32 candidate genes were identified using qRT-PCR-based expression profiling. Among them, a gene designated *OsRBSq3* (*Os03g0687400*), encoding a ribosome-inactivating protein family member that plays a role in defense mechanisms against necrotrophic pathogens, exhibited notable differential expression between Cheongcheong and Nagdong. These results provide new insights into the genetic architecture underlying brown spot resistance in rice and highlight *OsRBSq3* as a potential target for marker-assisted selection and functional studies.

**Supplementary Information:**

The online version contains supplementary material available at 10.1038/s41598-025-33890-y.

Rice (*Oryza sativa L.*) is a vital food crop worldwide, providing food for more than half of the global population. Its consistent production is essential to address growing food demands driven by population growth and changing dietary needs^1^. Among various challenges to rice cultivation, fungal diseases notably restrict both yield and grain quality. Brown spot disease, caused by the necrotrophic fungus *Bipolaris oryzae* (sexual stage: *Cochliobolus miyabeanus*), is widely distributed across rice-growing regions and can cause substantial yield reductions, occasionally reaching losses of 20–40% during severe outbreaks^2,3^. Environmental stresses such as nutrient deficiency, drought, or high temperatures can increase plant susceptibility to brown spot disease, often leading to more severe infections and reduced grain quality^4^. As global temperatures continue to rise, the risk and impact of brown spot are expected to intensify, making it a potentially more serious threat in the future^5^. While fungicides have been used to manage brown spot, concerns remain regarding environmental pollution, risks to human health, and the potential development of resistant pathogen strains^6^. Additionally, chemical control may not be a feasible option for many resource-limited farmers. Alternatively, breeding rice cultivars with inherent resistance represents a more sustainable, economical, and environmentally sound approach^7,8^. Achieving durable resistance necessitates resolving the genetic mechanisms governing brown spot resistance and establishing molecular markers that enable efficient marker-assisted selection.

The complexity of brown spot resistance lies in its quantitative inheritance and sensitivity to environmental variables, which complicates breeding efforts^9,10^. Conventional phenotypic selection has limitations due to these factors, emphasizing the value of molecular tools. Recent advances in DNA marker technology and high-throughput genotyping have enabled researchers to dissect such complex traits through QTL analysis^11^. QTL analysis enables the association of phenotypic variation with specific genomic regions, thereby allowing more precise selection of resistance traits. Several QTL analysis studies have demonstrated that brown spot disease in rice is quantitatively inherited and governed by multiple loci distributed across the genome^8,12,13^. A major and repeatedly detected QTL on chromosome 11, commonly referred to as *qBSR11*, has been identified in diverse populations and is considered a key contributor to resistance^8,14,15^. Additional QTLs have been reported on chromosomes 2, 6, 7, 9, and 12 from various resistant donors such as ‘Dawn’, ‘CH45’, ‘Tupa 121-3’, ‘Naba’, and ‘IR58’ ^8,12,13^. Ota et al. recently identified novel loci, including *qBSR6-kt*, and also confirmed previously reported QTLs located on chromosome 11, such as *qBSR11-kn*, *qBSR11-im*^15^. Despite these advances, there are still challenges that remain evident in the literature. Most reported QTL show population and environment specificity, which complicates straightforward marker deployment across breeding programs and underscores the importance of validation across backgrounds. Environmental fluctuations and pathogen diversity further affect the stability and expression of these QTLs, presenting challenges for their practical use in breeding. Therefore, continued QTL identification using diverse populations and pathogen isolates is essential to clarify the genetic architecture of brown spot resistance, identify loci with consistent effects, and uncover key genes that can enhance the efficiency of marker-assisted selection for durable resistance.

To further dissect the genetic basis of rice brown spot resistance, this study evaluated disease reactions in a doubled haploid population derived from a cross between the resistant cultivar ‘Cheongcheong’ and the susceptible cultivar ‘Nagdong’ (CNDH population) with a diverse collection of *Cochliobolus miyabeanus* isolates representing current field pathogen populations, to identify new resistance loci and candidate genes. By integrating QTL analysis with gene expression profiling, this study aimed to uncover genomic regions and genes associated with brown spot resistance. The findings are expected to provide molecular markers and gene targets useful for breeding programs focused on improving resistance to rice brown spot disease.

## Results

### Phenotypic assessment of rice brown spot in the CNDH population

**Fig. 1:**
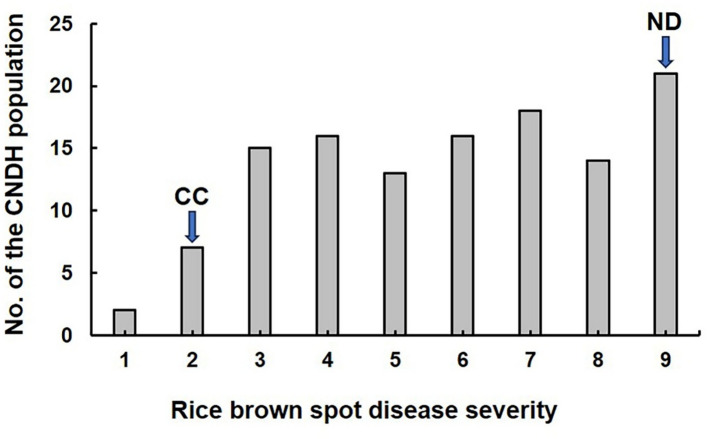
Frequency distribution of disease severity in the CNDH population and the parental lines Cheongcheong (resistant) and Nagdong (susceptible) in response to rice brown spot. CC, Cheongcheong; ND, Nagdong. Disease severity was scored based on the percentage of leaf area diseased: 1 = < 1%; 2 = 1–3%; 3 = 4–5%; 4 = 6–10%; 5 = 11–15%; 6 = 16–25%; 7 = 26–50%; 8 = 51–75%; 9 = 76–100%.

For QTL analysis, disease severity was assessed using a 0–9 numerical scale, recorded seven days after rice plants were inoculated with the pathogen responsible for rice brown spot. The average severity score across the 120 lines of the CNDH population was 5.8. As anticipated, the parental lines displayed contrasting responses: Cheongcheong exhibited resistance with a score of 2, while Nagdong was highly susceptible, scoring 9 (Fig. 1 and Supplementary Table S1).

### QTL analysis for rice brown spot resistance in the CNDH population

QTL analysis was conducted based on the disease severity data from the CNDH population. The analysis revealed a single significant QTL, designated *qRBS3*, located between markers RM15749 and RM15689 on chromosome 3, accounting for 21% of the phenotypic variation. The QTL had a LOD score of 2.68, with resistance attributed to the allele derived from Cheongcheong. This genomic region encompassing *qRBS3* was subsequently explored to identify candidate genes associated with resistance to rice brown spot (Supplementary Table S2).

### Identification of rice brown spot resistance-related genes within the RM15749–RM15689 marker region

**Fig. 2:**
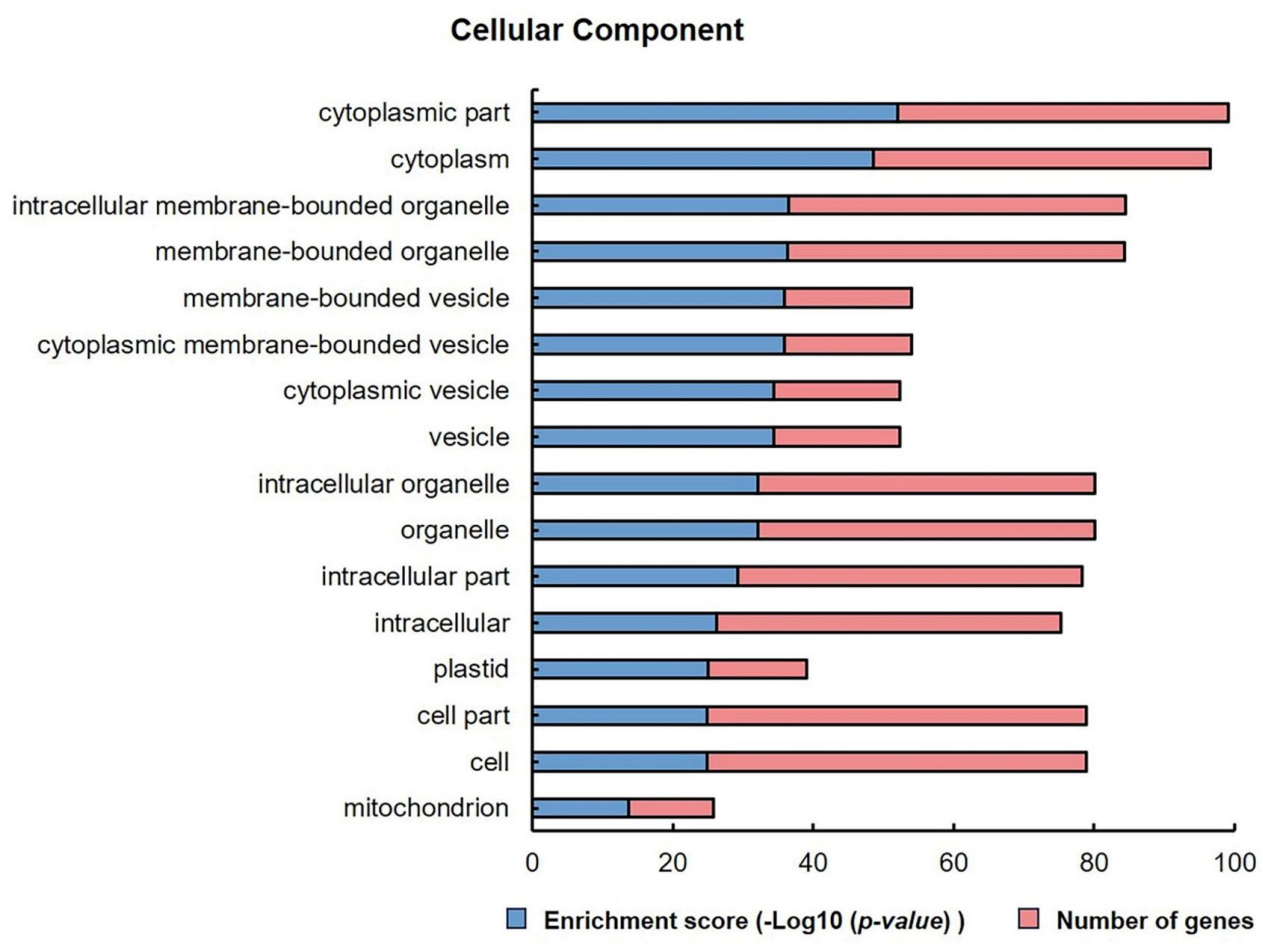
Gene ontology (GO) annotation of the QTL region between markers RM15749-RM15689 on chromosome 3. Sixteen GO terms associated with cellular components were identified.

To identify candidate genes involved in rice brown spot resistance, the genomic region between markers RM15749 and RM15689 on chromosome 3 was examined using the RiceXPro and Rice Annotation Project databases. A total of 69 genes were identified based on their annotation within the region RM15749- RM15689 from 27,323,614 bp to 28,098,640 bp (Supplementary Table S3). Functional classification of these genes was performed using agriGO, a gene ontology (GO) analysis tool. The analysis revealed several significantly enriched GO terms, all categorized under cellular component. The five top significant GO terms included cytoplasmic part, cytoplasm, intracellular membrane-bounded organelle, membrane-bounded organelle, and membrane-bounded vesicle (Fig. 2). These categories are closely associated with the plant’s defense system. In particular, the cytoplasm and membrane-bounded organelles serve as key sites for signal transduction, biosynthesis of defense-related compounds, and activation of immune responses. Membrane-bounded vesicles also play a role in the intracellular transport and secretion of pathogenesis-related proteins, which are essential for responding to pathogen attacks. The enrichment of these GO terms suggests that genes within this region may contribute to resistance against rice brown spot by regulating crucial cellular processes involved in the plant’s innate immune response.

### Relative expression levels of rice brown spot resistance related genes

**Fig. 3:**
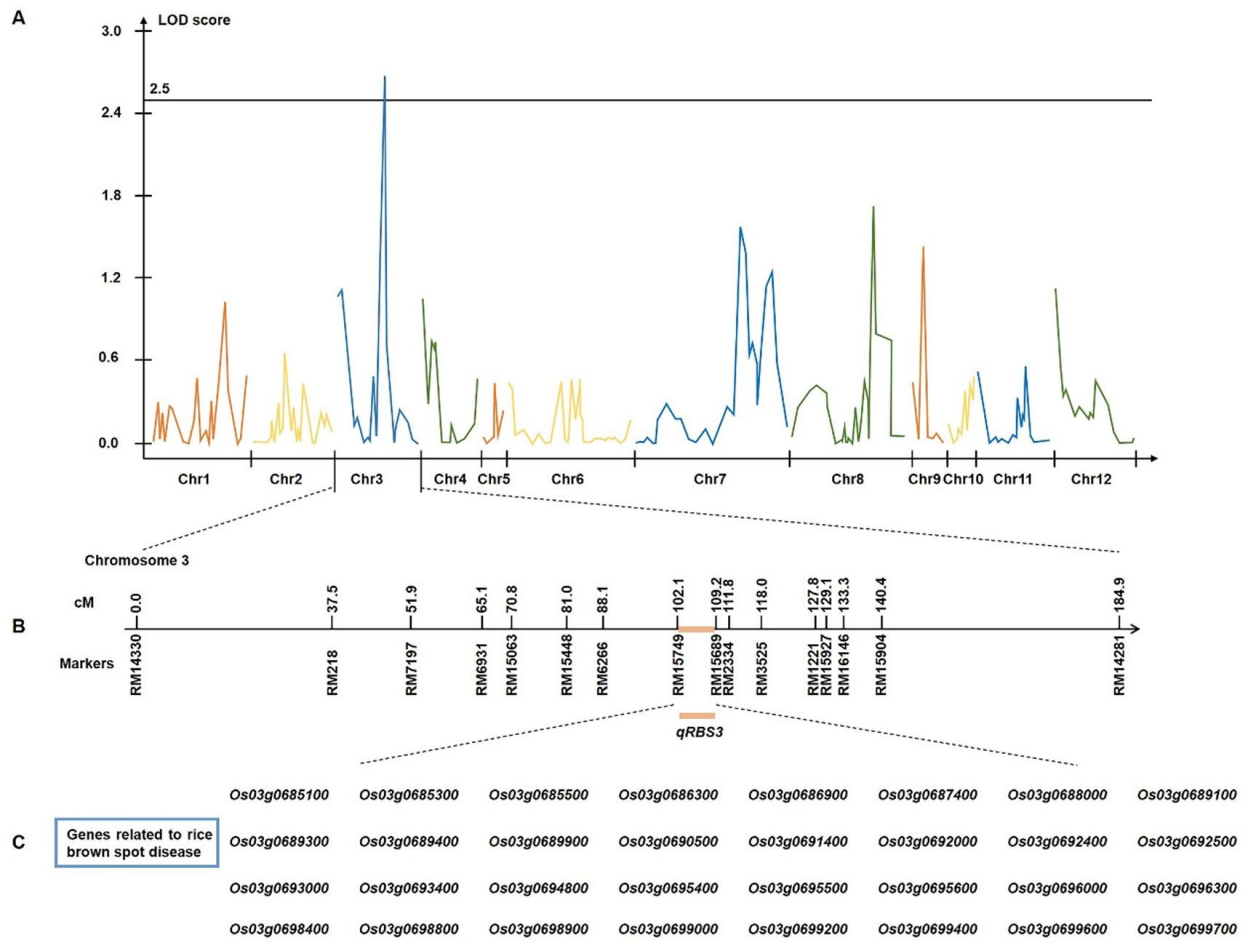
QTL analysis and physical mapping of the locus associated with rice brown spot resistance. (**A**) QTL analysis showing a peak with an LOD score of 2.68 on chromosome 3. (**B**) The target interval between markers RM15749 and RM15689 on chromosome 3. (**C**) Thirty-two candidate genes related to rice brown spot resistance were identified within the RM15749–RM15689 interval.

**Fig. 4:**
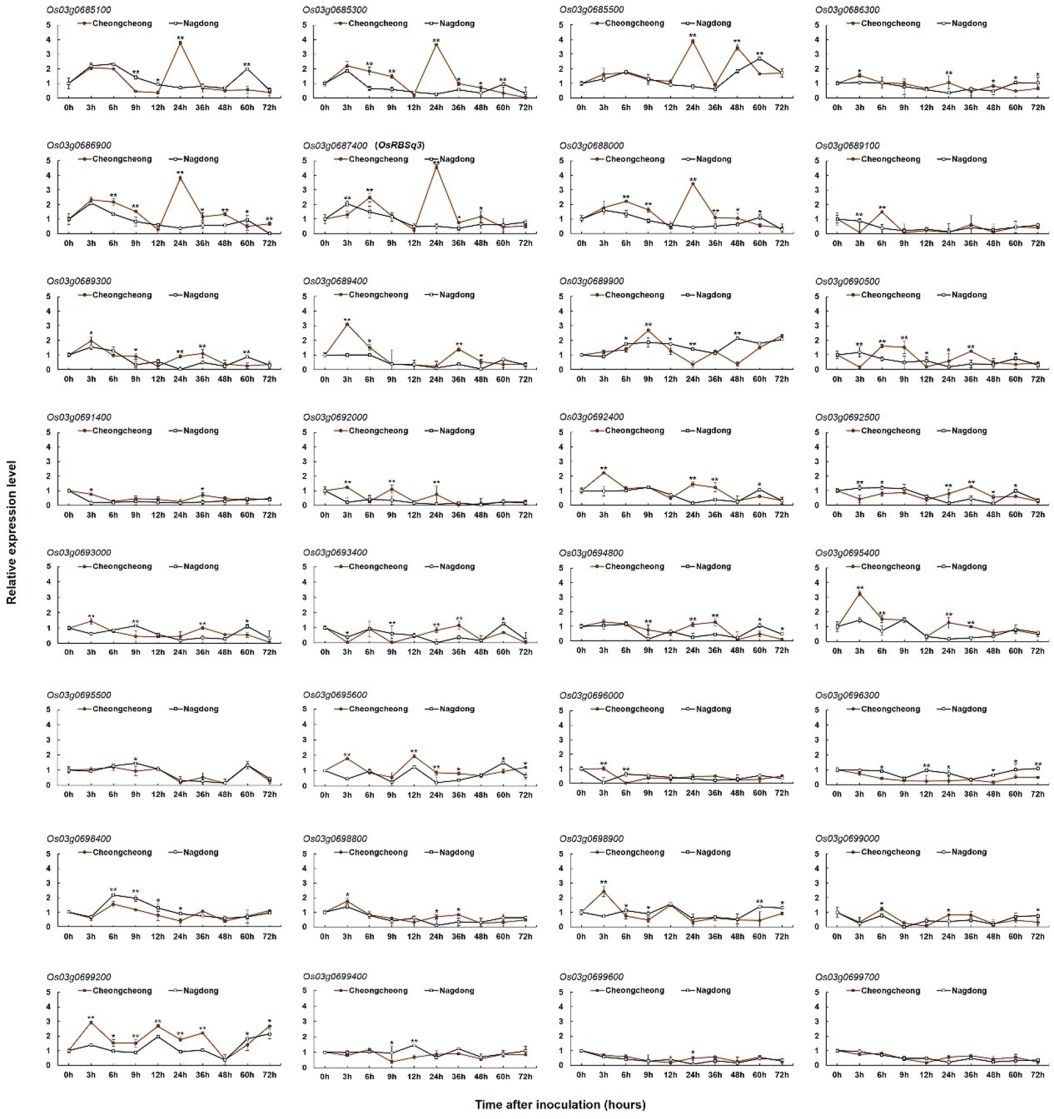
Comparison of the expression of 32 selected rice brown spot resistance-related genes identified within the QTL analysis. Expression levels were analyzed in the resistant line Cheongcheong and the susceptible line Nagdong. Data represent mean ± standard deviation (*n* = 3). Asterisks indicate significant differences between cultivars (*=*P* < 0.05, **=*p* < 0.01; two-tailed Student’s t test).

**Fig. 5:**
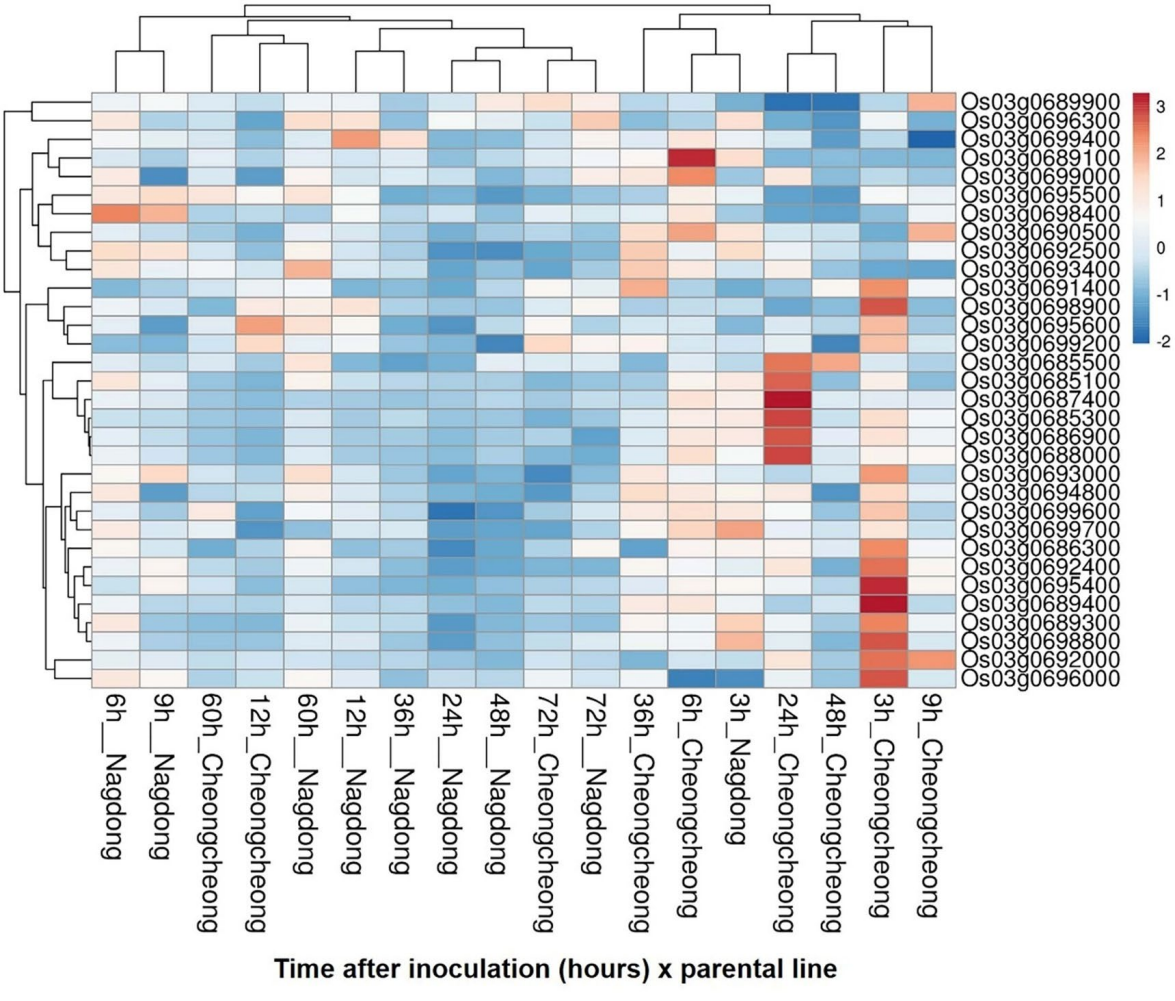
Heatmap of gene expression patterns in Cheongcheong and Nagdong across different time points. Rows represent genes, and columns represent samples under various conditions. Both genes and samples were clustered based on principal component analysis (PCA) distance. The color scale indicates relative expression levels, with red representing higher expression and blue representing lower expression. The heatmap was generated using the ClustVis (version 2.0; https://biit.cs.ut.ee/clustvis/) based on the gene expression data.

Among the 69 genes located within the target marker interval RM15749–RM15689 on chromosome 3, 32 were selected for further analysis based on gene annotation (Fig. 3). To investigate their expression patterns following rice brown spot inoculation, Real-time PCR (qRT-PCR) analysis was conducted on the two parental rice lines: the resistant cultivar Cheongcheong and the susceptible cultivar Nagdong. Samples were collected at 0, 3, 6, 9, 12, 24, 36, 48, 60, and 72 h post-inoculation, and gene-specific primers used in the analysis are listed in Supplementary Table S4. Several genes, including *Os03g0686300*, *Os03g0689300*, *Os03g0689400*, *Os03g0692000*, *Os03g0692400*, *Os03g0693000*, *Os03g0694800*, *Os03g0695400*, *Os03g0696000*, *Os03g0698800*, *Os03g0699600*, and *Os03g0699700*, displayed similar expression patterns in both cultivars. This suggests that these genes may be involved in general stress responses rather than cultivar-specific defense mechanisms. In contrast, *Os03g0685100*, *Os03g0685300*, *Os03g0685500*, *Os03g0686900*, *Os03g0687400*, and *Os03g0688000* show a pronounced upregulation in Cheongcheong, particularly between 24- and 48-hours post-inoculation (Figs. 4 and 5). This expression pattern indicates a potential role in the early defense response to *Cochliobolus miyabeanus* infection. Notably, *Os03g0687400*, which encodes a ribosome-inactivating protein, exhibited the most significant differential expression between the two parental rice lines. In Cheongcheong, the gene was strongly induced as early as 6 h post-inoculation and peaked at 24 h with approximately a 4.6-fold increase relative to the 0-hour time point. In contrast, Nagdong displayed a modest induction at 3 h (~ 2-fold), followed by a continuous decline with no significant upregulation at later time points. These findings suggest that *Os03g0687400*, named as *OsRBSq3*, may play a critical role in the defense response of the resistant cultivar and could contribute to brown spot resistance in rice.

### Computational analysis of the candidate gene *OsRBSq3*

**Fig. 6:**
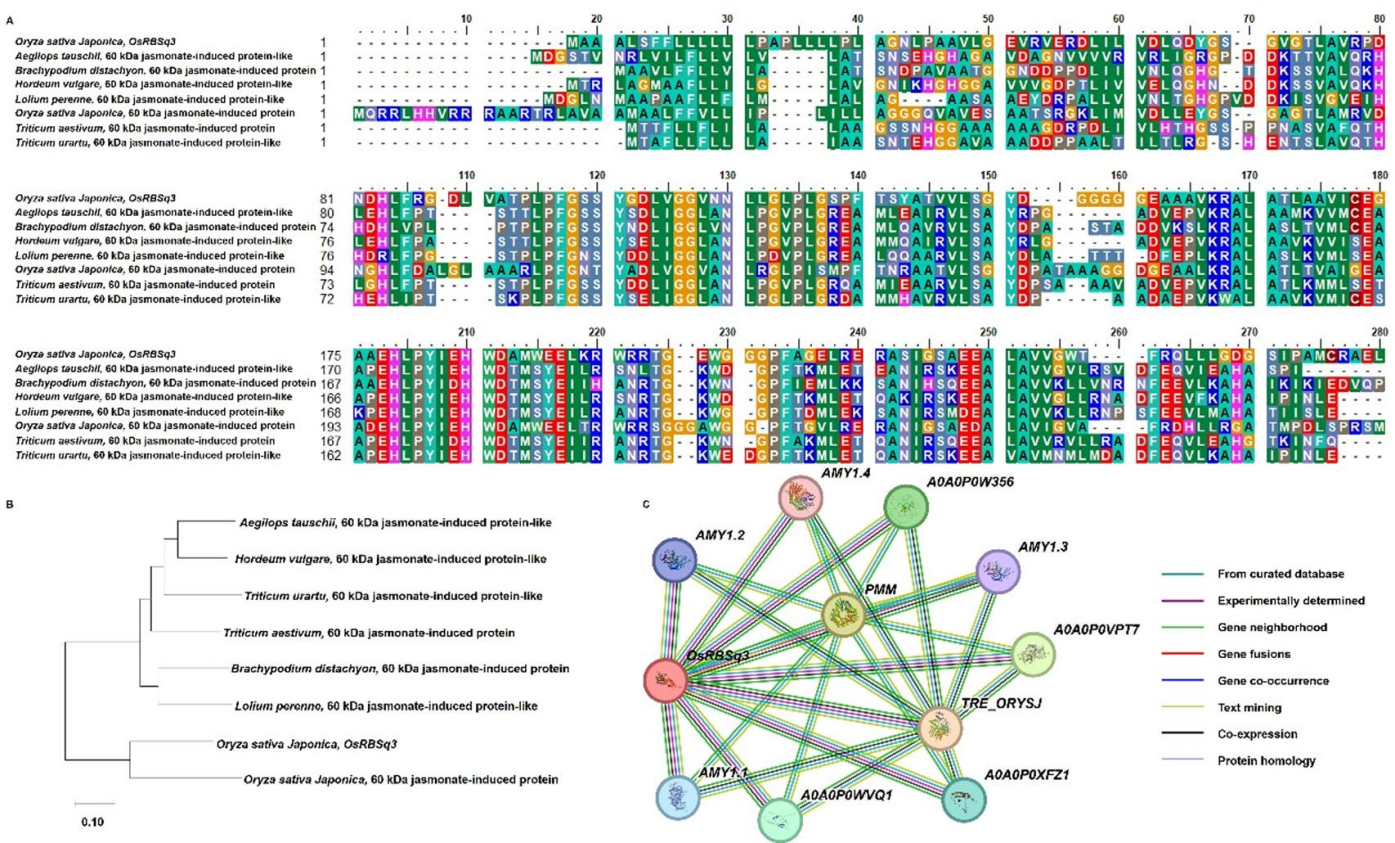
Sequence analysis of *OsRBSq3*. (**A**) Proteins with high sequence similarity to *OsRBSq3* were identified in *Aegilops tauschii*, *Brachypodium distachyon*, *Hordeum vulgare*, *Lolium perenne*, *Oryza sativa ssp. japonica*, *Triticum aestivum*, and *Triticum Urartu*. Multiple sequence alignments was performed using BioEdit (version 7.0; https://bioedit.software.informer.com/) (**B**) Phylogenetic analysis of *OsRBSq3* and its homologs was conducted using the maximum likelihood method with 1,000 bootstrap replicates in MEGA (version 12; https://www.megasoftware.net/) (**C**) Predicted protein–protein interactions of *OsRBSq3* were analyzed using the STRING database (version 12; https://string-db.org/), showing associations with 10 proteins: TRE_ORYSJ, PMM, A0A0P0VPT7, A0A0P0W356, A0A0P0WVQ1, A0A0P0XFZ1, AMY1.1, AMY1.2, AMY1.3, and AMY1.4.

The candidate gene *OsRBSq3* was identified as being associated with rice brown spot resistance at the seedling stage through QTL analysis of the 120 CNDH population. Sequence similarity searches using NCBI BLAST indicated that the predicted protein encoded by *OsRBSq3* shares a high degree of homology with a 60 kDa jasmonate-induced protein reported in several species, including *Aegilops tauschii*, *Brachypodium distachyon*, *Hordeum vulgare*, *Lolium perenne*, and *Oryza sativa ssp. japonica*, *Triticum aestivum*, and *Triticum Urartu* (Fig. 6A). Phylogenetic analysis further confirmed the close evolutionary relationship of *OsRBSq3* with homologous proteins in these species (Fig. 6B). Functional interaction predictions suggested that *OsRBSq3* may be associated with ten putative partner genes, including TRE_ORYSJ (glycosyl hydrolase 37 family), PMM (phosphomannomutase), A0A0P0VPT7 (glycosyl hydrolase 13 family), A0A0P0W356 (Os03g0756700 protein), A0A0P0WVQ1 (Os06g0286310 protein), A0A0P0XFZ1 (Os08g0391501 protein), AMY1.1 (glycosyl hydrolase 13 family), AMY1.2 (α-amylase isozyme 3 A), AMY1.3 (α-amylase isozyme 3D), and AMY1.4 (α-amylase isozyme 3E, glycosyl hydrolase 13 family) (Fig. 6C).

## Discussion

Rice remains the major staple crop in Korea, and its productivity is increasingly constrained by brown spot disease, which affects plants from the seedling stage through maturity and often results in severe yield reductions^15,16^. Although fungicide application can mitigate disease incidence, its effectiveness is limited by restrictions on chemical use and rising concerns over eco-friendly agricultural practices^17^. Moreover, climate change has extended the duration of favorable conditions for pathogen development, further intensifying disease pressure^5^. While deploying resistant cultivars offers a more sustainable strategy, breeding for durable resistance is complicated by the high variability of the pathogen population. Therefore, identifying and utilizing genetic resistance is considered the most practical approach, as it reduces production costs and minimizes dependence on chemical control.

In our study, using the CNDH population derived from a cross between the resistant cultivar ‘Cheongcheong’ and susceptible cultivar ‘Nagdong’, we identified a single moderate-effect QTL, designated *qRBS3*, on chromosome 3 between SSR markers RM15749 and RM15689. This QTL accounted for 21% of phenotypic variation (LOD 2.68), with the resistance allele contributed by the parent Cheongcheong. Previous studies have reported brown spot resistance QTLs mainly on chromosomes 2, 3, 6, 7, 9, and 11, particularly in populations derived from crosses involving CH45, Koshihikari, or the American cultivar Dawn^8,13,15^. In the present study, an additional significant genomic region *qRBS3* was detected on chromosome 3 within the physical interval of 27,323,614 bp and 28,098,640 bp. This region *qRBS3* partially overlaps with the previously reported locus *qBSR3.2* (25,722,435 bp-29,085,775 bp) described by Ota et al., which is associated with brown spot disease but is independent of heading date^18^. Such an overlap indicates that the region detected in the CNDH population may correspond to the same or a tightly linked locus contributing to brown spot resistance, offering the potential to enhance resistance without compromising agronomic performance. The detection of *qRBS3* on chromosome 3 further supports the importance of this chromosome as a key target for future resistance breeding strategies in rice. In addition to identifying significant QTLs for brown spot resistance, our study also examined the genes located within the target marker interval to highlight potential candidate genes underlying the detected locus. Further investigation of the *qRBS3* interval, we annotated 69 candidate genes, with GO enrichment highlighting components such as cytoplasm, intracellular membrane-bounded organelle, and vesicle cellular sites critical for signal transduction, defense compound biosynthesis, and pathogenesis-related protein trafficking. This aligns with a recognized model where pathogen perception, signaling, and host defense execution involve intracellular vesicle trafficking and membrane-associated processes^19–21^. Furthermore, we selected 32 candidate genes examined via qRT-PCR, several were markedly induced in the resistant Cheongcheong following inoculation, especially between 24 and 48 h. Among them, *Os03g0687400*, encoding a ribosome-inactivating protein, showed a 4.6-fold increase in expression at 24 h in Cheongcheong, compared to only transient induction in Nagdong. We designated this gene *OsRBSq3* and proposed it as a strong candidate effector of *qRBS3*-mediated resistance. Ribosome-inactivating proteins represent a broad gene family in plants, with more than 30 members reported in cereals, often divided into type I (single-chain) and type II (linked to lectin-like domains)^4,13^. These proteins are well-known for their rRNA N-glycosidase activity, which acts as a “last line of defense” against invasive pathogens by permanently deactivating ribosomes and stopping protein production^22^. Although ribosome-inactivating protein-like genes in rice have been linked to stress adaptation in the past, their function in brown spot resistance has not yet been elucidated^13^.

Phylogenetically, *OsRBSq3* clusters with jasmonate-induced 60 kDa proteins across species such as wheat, barley, and Brachypodium, many of which are inducible by pathogen infection^23^. This evolutionary conservation suggests that *OsRBSq3* belongs to a defense-related ribosome-inactivating protein subfamily tightly linked to jasmonic acid (JA) signaling. The STRING analysis further linked *OsRBSq3* to glycosyl hydrolases (GH) 13 and GH 37, as well as α-amylases, enzymes often activated during pathogen invasion. Glycosyl hydrolases not only participate in carbohydrate metabolism but also contribute to cell-wall remodeling and the release of oligosaccharide-derived damage signals, which, in turn, can trigger immune responses^5^. The *OsRBSq3*-centered network may therefore be a synergistic defense cluster where GH family members fortify host barriers and trigger metabolic responses, while ribosome-inactivating protein activity restricts fungal colonization. This argument is supported by the similarities with other brown spot resistance genes. For instance, *OsFBN6*, a fibrillin protein involved in plastid lipid metabolism, increases brown spot resistance through JA signaling^17^. While *OsFBN6* and *OsRBSq3* differ in mechanistic roles and chromosomal location, both are inducible components of JA-associated defense, suggesting that multiple defense modules converge on JA-regulated networks. Similarly, GWAS-based identification of *OsExo70F3* and *OsBSR820* on chromosome 4 highlights vesicle trafficking and transcriptional regulation as critical defense components^16^. Together, these studies suggest that while brown spot resistance involves diverse gene families (ribosome-inactivating proteins, fibrillin, exocyst components, transcriptional regulators), their convergence on hormone signaling and cellular defense architecture points to shared resistance frameworks. Our results suggest that *qRBS3* likely confers quantitative, partial resistance mediated by early induction of *OsRBSq3*, promoting a rapid defense response, whereas other QTLs like those mapped to chromosomes 2, 9, and 11 may incorporate different defense strategies, such as structural barriers, detoxification, or signaling modulation. This highlights the polygenic complexity of brown spot resistance and the need for gene pyramiding to achieve durable field-level resistance. Pyramiding our newly identified locus *qRBS3* with previously reported loci such as *qBSR11*, together with the candidate gene *OsRBSq3*, may provide complementary resistance mechanisms derived from multiple defense-related gene families. Such combinations have the potential to strengthen resistance durability in breeding programs. From a practical standpoint, markers flanking *qRBS3* can be incorporated into marker-assisted selection schemes. Although *qRBS3* confers a moderate effect on resistance, this characteristic makes it particularly suitable for stacking with major and stable QTLs like *qBSR11*, which has demonstrated consistent performance across diverse environments^24^. Integrating these loci is expected to enhance brown spot resistance without negatively impacting key agronomic traits, including heading date and yield.

Overall, our study introduces a novel brown spot resistance locus, *qRBS3* on chromosome 3, with a compelling candidate gene *OsRBSq3* encoding a ribosome-inactivating protein. This locus adds a new dimension to the genetic architecture of brown spot resistance and underscores the complexity of quantitative defense. The gene family context further strengthens the mechanistic plausibility of *OsRBSq3*, given the conserved defensive functions of ribosome-inactivating proteins, glycosyl hydrolases, and JA-induced proteins across cereals. Functional validation via CRISPR knockout or overexpression, coupled with multi-omics to define its interaction partners, will be the next crucial steps. Moreover, integrating *qRBS3* into marker-assisted selection pipelines alongside other family-specific resistance genes could accelerate the development of cultivars with broad, durable resistance to brown spot.

## Materials and methods

### Plant material

In this study, a doubled haploid population consisting of 120 lines was produced by anther culture using an F_1_ population derived from a cross between *O. sativa spp. Indica* Cheongcheong and *O. sativa spp. Japonica* Nagdong was used. Since the CNDH population establishment in 2010, the CNDH population has been continuously maintained and cultivated for over a decade in the paddy fields of Kyungpook National University, located in Gunwi-gun, Gyeongsangbuk-do, Republic of Korea (36°11′ N, 128°64′ E), where it has also served as a bridge parent in breeding programs^25,26^. Cheongcheong exhibits strong resistance to major rice diseases and pests, whereas Nagdong is generally susceptible^27,28^.

### Isolation of the *Cochliobolus miyabeanus*

Six *Cochliobolus miyabeanus* isolates 23CM4, 23CM5, 23CM6, 23CM11, 23CM13, and 23CM14 were collected in 2023 from rice fields in Sancheong-gun, Gyeongsangnam-do (23CM4); Gunsan-si, Jeollabuk-do (23CM5 and 23CM6); Sacheon-si, Gyeongsangnam-do (23CM11 and 23CM13); and Goseong-gun, Gyeongsangnam-do (23CM14). These isolates, identified as the most virulent strains in our laboratory, were used in the present study. Each isolate was obtained as a single-spore culture from lesions on infected rice leaves. Infected leaf segments were cut and surface-sterilized in 70% ethanol for 30 s, followed by rinsing in sterile distilled water. Excess moisture was removed using sterile filter paper. Lesion tissues were then placed on water agar medium (1.5% [w/v] agar powder) and incubated at room temperature for 24 h. Spores developing on the lesions were collected under a microscope using a sterile loop and transferred to potato dextrose agar (PDA; MB-P1120, Kisanbio, Seoul, Korea), where they were cultured for 5 days. Sterile Advantec paper discs (Advantec Toyo Kaisha Ltd.) were placed on the 5-day-old PDA cultures and incubated at 26 °C until fully colonized by mycelia. The colonized discs were carefully transferred to empty Petri dishes and air-dried at room temperature for 20–25 days. Once fully dried, the paper discs were stored in Eppendorf tubes at − 80 °C for long-term preservation.

### Inoculum preparation and inoculation

Conidia of six *Cochliobolus miyabeanus* isolates (Supplementary Figure S1) were harvested from 10-day-old cultures grown on PDA using 250 ppm of Tween 20 (CAS:9005-64-5). The conidial suspension was adjusted to a concentration of 1 × 10^5^ conidia/mL. The CNDH population, along with the parental lines Cheongcheong and Nagdong, was inoculated at the four-leaf stage by spraying the conidial mixture until the leaf surfaces were uniformly covered with fine droplets. Following inoculation, plants were incubated in a dew chamber at 26 °C with 100% relative humidity for 16 h to facilitate fungal penetration. Subsequently, plants were transferred to a greenhouse at 25 to 30 °C and approximately 80% relative humidity for disease development. Disease severity was assessed based on the percentage of infected leaf area and lesion types, following the IRRI Standard Evaluation System for rice^29^. Symptoms were scored at 7 days post-inoculation using a 0–9 scale, where 0 indicates no symptoms, and 9 corresponds to 76%-100% leaf area affected. Representative infected leaves were photographed. Each infection assay was independently repeated at least three times. When phenotypic variation occurred within a CNDH line, the disease severity score was defined as the highest score recorded among a minimum of three plants in that line.

### QTL mapping population and statistical analysis

A total of 788 SSR markers were initially screened for polymorphism in the construction of the CNDH population genetic map. Among them, 423 markers exhibited polymorphic patterns, and 222 co-dominant markers confirmed through PCR amplification were selected for map development. The resulting genetic linkage map spanned 2121.7 centiMorgan (cM), with an average interval of 10.6 cM between adjacent markers^25,30^. Marker placement and linkage analysis were conducted using Mapmaker version 3.0, and the markers were distributed evenly across all 12 chromosomes of rice^31^. Subsequent QTL analysis was performed to identify genomic regions associated with rice brown spot disease. The significance threshold for QTL detection was determined using 1000 permutation tests at a 0.05 significance level in Windows QTL Cartographer version 2.5. The analysis employed composite interval mapping (CIM) through Windows QTL Cartographer version 2.5^32^. A minimum LOD (logarithm of odds) threshold of 2.5 was applied to ensure statistical confidence in QTL detection^32^. Input data included genotypic profiles, marker distances, the number of chromosomes, and phenotypic measurements for the target traits. The naming of QTLs followed the standardized nomenclature system proposed by McCouch and Doerge^33^. Histograms were generated using Microsoft Excel (Microsoft Excel 2019) to visualize the distribution of phenotypic data. The Excel built-in chart tools were used to organize and display the frequency of disease severity scores across the evaluated population.

### Candidate gene analysis and bioinformatics tools

Candidate genes within the identified QTL regions were screened using data from RiceXpro^34^ and the Rice Annotation Project Database^35^. Open reading frames (ORFs) located near simple sequence repeat (SSR) markers were identified and annotated based on predicted gene functions. Gene ontology (GO) enrichment analysis was performed using the agriGO (https://systemsbiology.cau.edu.cn/agriGOv2/) (Job ID: 708740574.1) (accessed on 07 December 2025) to characterize the functional categories of the associated genes^36^. To identify homologous gene sequences, sequence similarity searches were conducted using NCBI BLAST, and multiple sequence alignments were performed using BioEdit version 7.0 (https://bioedit.software.informer.com/)^37^. A phylogenetic tree was generated using the maximum likelihood method in MEGA 12 (https://www.megasoftware.net/) with 1000 bootstrap replicates to evaluate evolutionary relationships^38^. Additionally, protein-protein interaction networks were analyzed using the STRING (https://string-db.org/) (accessed on 07 December 2025) database (version 12) to explore functional associations among the candidate proteins^39^.

### Expression analysis of genes associated with resistance to rice brown spot

To explore the molecular mechanisms underlying resistance to rice brown spot, the expression levels of candidate genes located within QTL regions associated with resistance were analyzed using quantitative real-time PCR (qRT-PCR). Two parental lines, Cheongcheong (resistant) and Nagdong (susceptible), were selected for expression profiling. Leaf samples were collected at multiple time points post-inoculation (0, 3, 6, 9, 12, 24, 36, 48, 60, and 72 h) for RNA extraction. Total RNA was extracted from pooled leaf tissue collected from at least three independent plants, which together constituted one biological replicate, and for each pooled biological sample, three technical replicates were performed to ensure reproducibility of the expression measurements. RNA extraction was performed using the RNeasy Mini Kit (QIAGEN, Hilden, Germany) according to the manufacturer’s protocol and adjusted to a concentration of 100 ng/µL with RNase-free water. First-strand cDNA synthesis was carried out using the iScript™ cDNA Synthesis Kit (catalog number 1708890; Bio-Rad Laboratories, Ann Arbor, MI, USA) with 100 ng of RNA template. qRT-PCR was performed in a 20 µL reaction mixture using iTaq™ Universal SYBR^®^ Green Supermix (Catalog number 1725120; Bio-Rad Laboratories). The thermal cycling protocol consisted of an initial denaturation at 95 °C for 3 s, followed by 40 cycles of annealing and extension at 60 °C for 20 s. Gene expression levels were normalized to the internal reference gene *OsActin*, and three technical replicates of qRT-PCR were performed. Mean expression values and standard deviations were calculated to assess gene expression dynamics over time. A heatmap of gene expression data was generated using the ClustVis web tool (https://biit.cs.ut.ee/clustvis/) (accessed on 07 December 2025)^40^. The input data consisted of normalized gene expression values. Principal component analysis (PCA)-based clustering was applied to visualize patterns of gene expression across samples. The data matrix was log-transformed, and both rows (genes) and columns (samples and conditions) were clustered using the default setting. The heatmap was used to identify expression patterns associated with plant disease response.

## Conclusion

Rice brown spot poses a serious threat to yield stability and food supply in many rice-growing regions. In the present study, QTL analysis was conducted for the CNDH population with six *Cochliobolus miyabeanus* isolates collected from diverse areas of South Korea. We detected a new locus on chromosome 3, designated *OsRBSq3*, that accounted for 21% of the phenotypic variation in resistance. Sequence analysis revealed that *OsRBSq3* is homologous to the ribosome-inactivating protein family, suggesting its potential role in the defense signaling mechanism. This newly identified locus provides a valuable target for further functional validation and can be utilized in marker-assisted selection to develop rice cultivars with improved resistance to brown spot. Incorporating *OsRBSq3* into rice improvement programs may enhance resistance to brown spot, thereby improving crop resilience and contributing to food security in regions where rice is a dietary staple.

## Supplementary Information

Below is the link to the electronic supplementary material.

Supplementary Material 1

## Data Availability

Data is provided within the manuscript or supplementary information files.
