# Supplementary material for: Unveiling key genetic loci and candidate genes for brown spot disease resistance in rice based on QTL analysis
Source: Sci Rep. 2026 Jan 29;16:3925. doi: 10.1038/s41598-025-33890-y (PMC12855810; doi:10.1038/s41598-025-33890-y)
Supplement: Supplementary file 1 — Supplementary Material 1 [file 41598_2025_33890_MOESM1_ESM.docx]

**Unveiling Key Genetic Loci and Candidate Genes for Brown Spot Disease Resistance in Rice Based on QTL Analysis**

Dan–Dan Zhao^1†^, Hyunjung Chung^1†^, Muhammad Farooq^1^, Nam–Gu Kim^1^, Soo Yeon Choi^1^, Shinhwa Kim^1^, Sang–Min Kim^1^, Jang-Ho Lee^1^, Xiao–Xuan Du^2,3*^, and Kyung–Min Kim^3,4*^

^1^ Crop Environment Research Division, National Institute of Crop and Food Science, Rural Development Administration, Wanju 55365, Korea

^2^ College of Marine and Bioengineering, YanCheng Institute of Technology, 211 Jianjun East Road, Yangcheng City, Jiangsu Province, 224051, China

^3^ Coastal Agriculture Research Institute, Kyungpook National University, Daegu 41566, Korea

^4^ Department of Applied Biosciences, Kyungpook National University, Daegu 41566, Korea

^*^Corresponding authors: X.–X. Du; Haobingshuaike@hotmail.com, and K.–M. Kim;

kkm@knu.ac.kr

^†^D.–D. Zhao and H. Chung contributed equally.

Dan–Dan Zhao: zhaodandan@korea.kr; ORCID 0000–0001–9375–3981.

Hyunjung Chung: chunghyunjung@korea.kr; ORCID 0000–0001–7258–6977.

Muhammad Farooq: mfarooqsr@gmail.com; ORCID 0000–0002–4917–4402.

Nam–Gu Kim: soybreeding@gmail.com;

Soo Yeon Choi: choisy99@korea.kr; ORCID 0000-0001-5758-5749.

Shinhwa Kim: sinhwa0103@korea.kr; ORCID 0009-0009-4763-7299.

Sang–Min Kim: kimsangmin@korea.kr; ORCID 0000-0003-2187-9336.

Jang-Ho Lee: junggl2@korea.kr

Xiao–Xuan Du: Haobingshuaike@hotmail.com; ORCID 0000–0003–0294–6740.

Kyung–Min Kim: kkm@knu.ac.kr; ORCID 0000–0003–4812–6297.


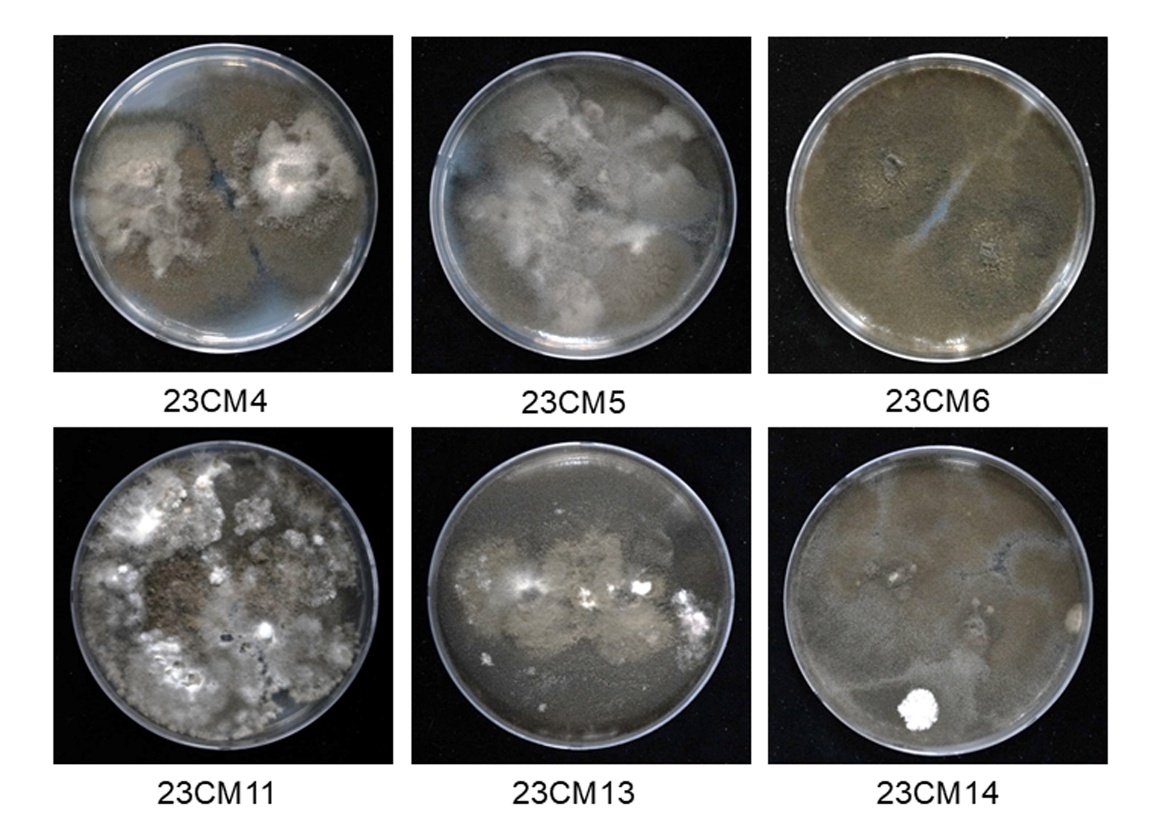


**Supplementary Figure S1.** Six *Cochliobolus miyabeanus* isolates collected from different regions of Korea in 2023.

**Supplementary Table S1.** Disease severity scores of the CNDH population in response to rice brown spot disease.**
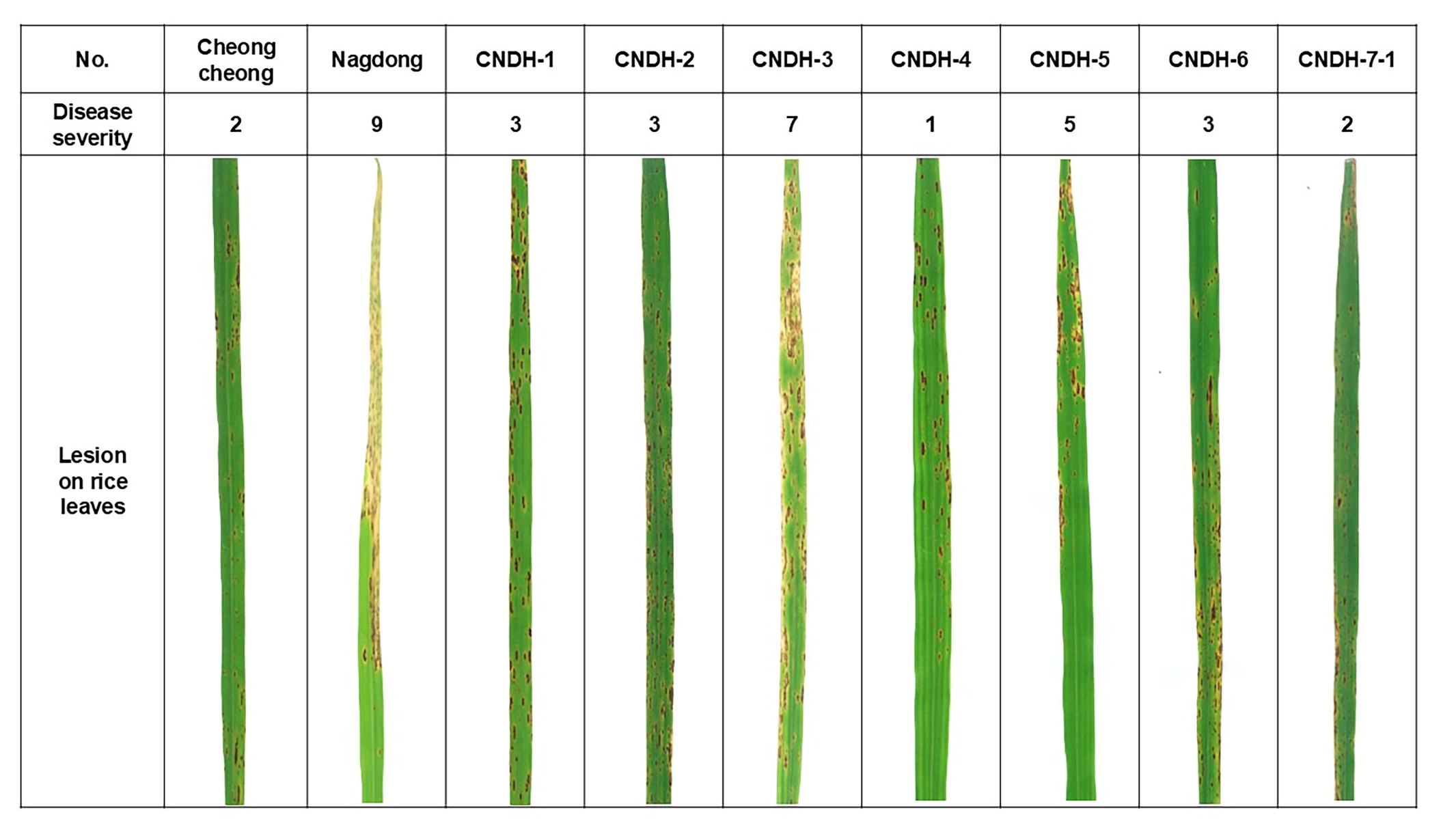
**

**
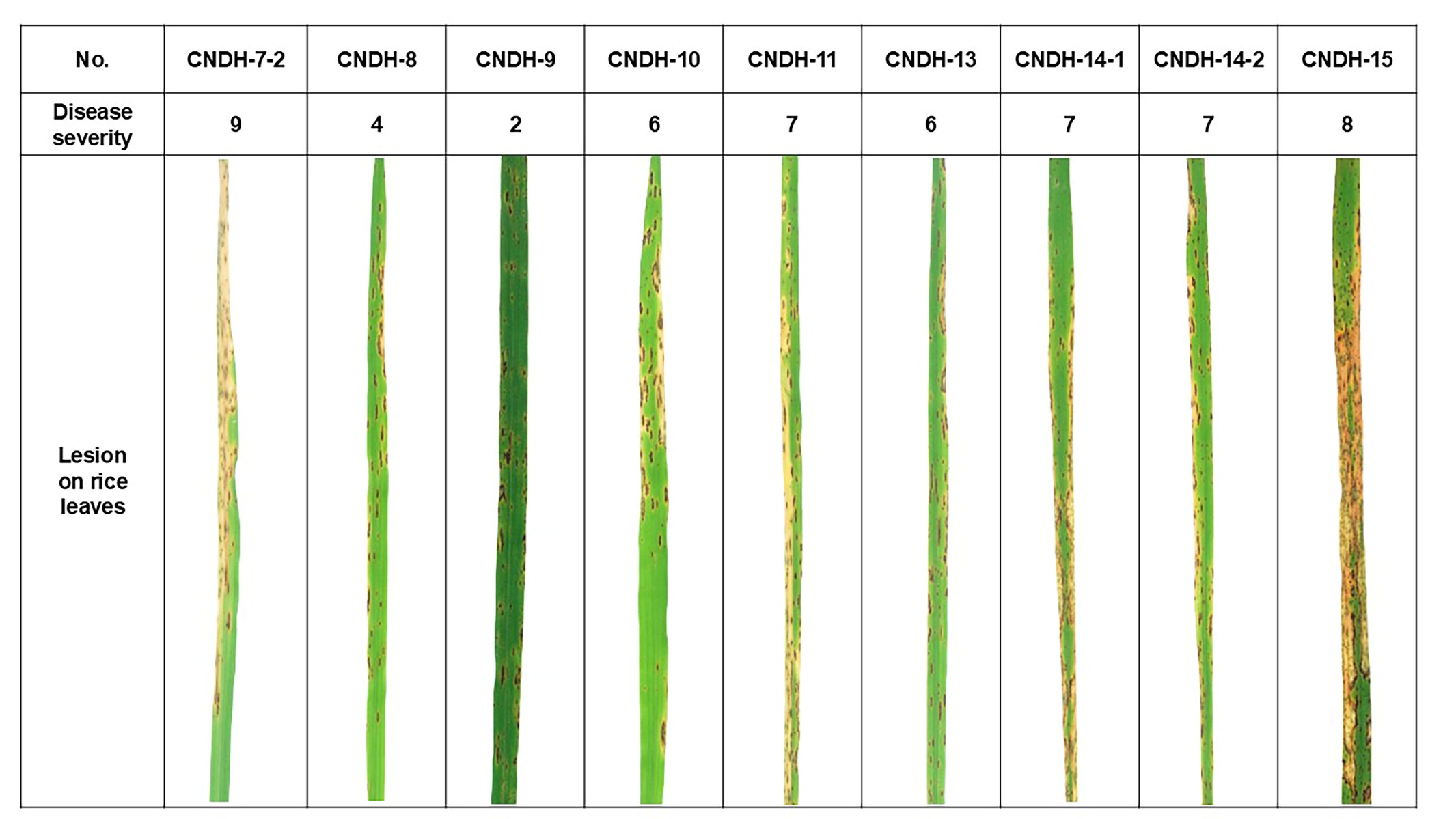
**

**
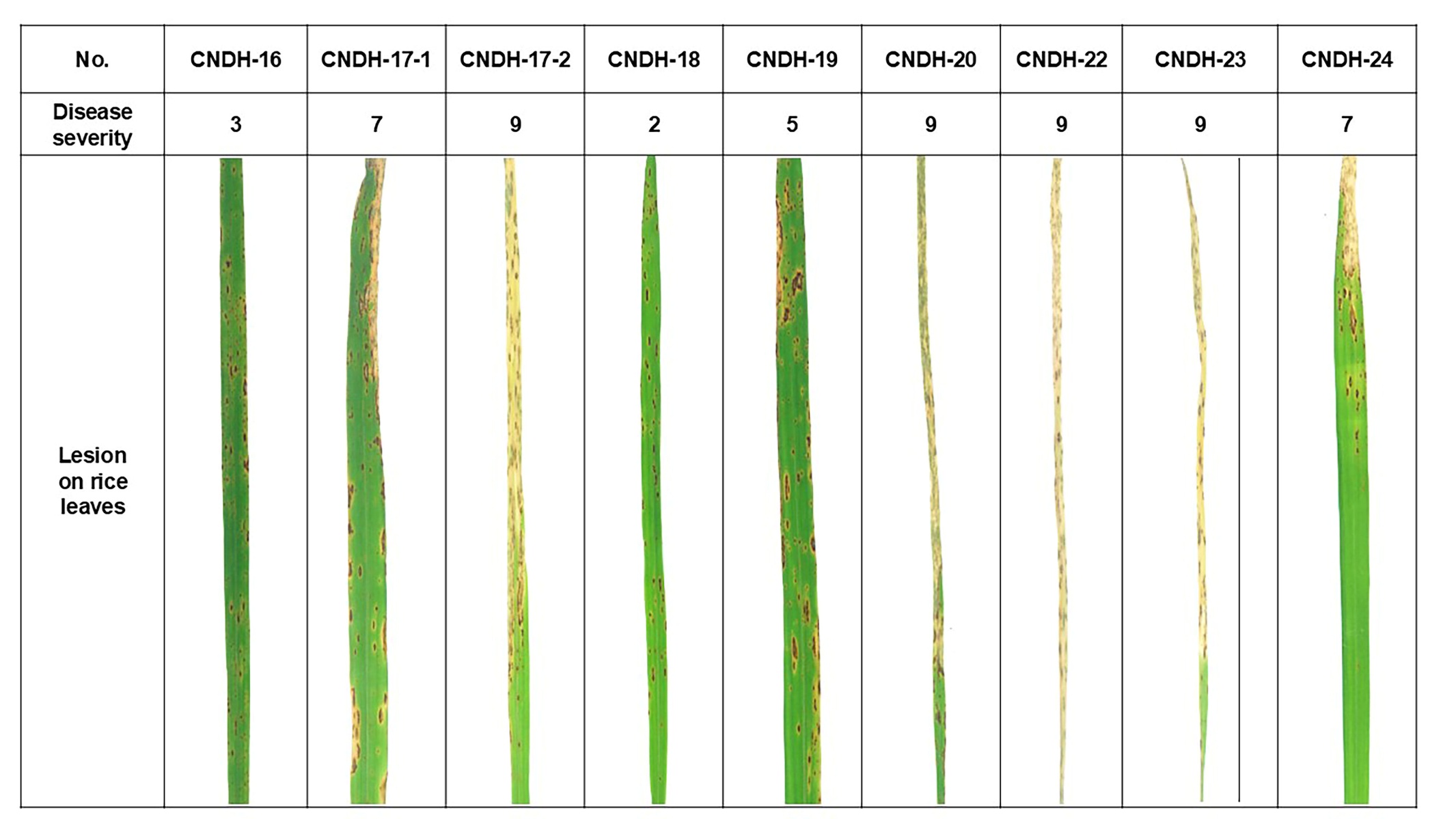
**

**
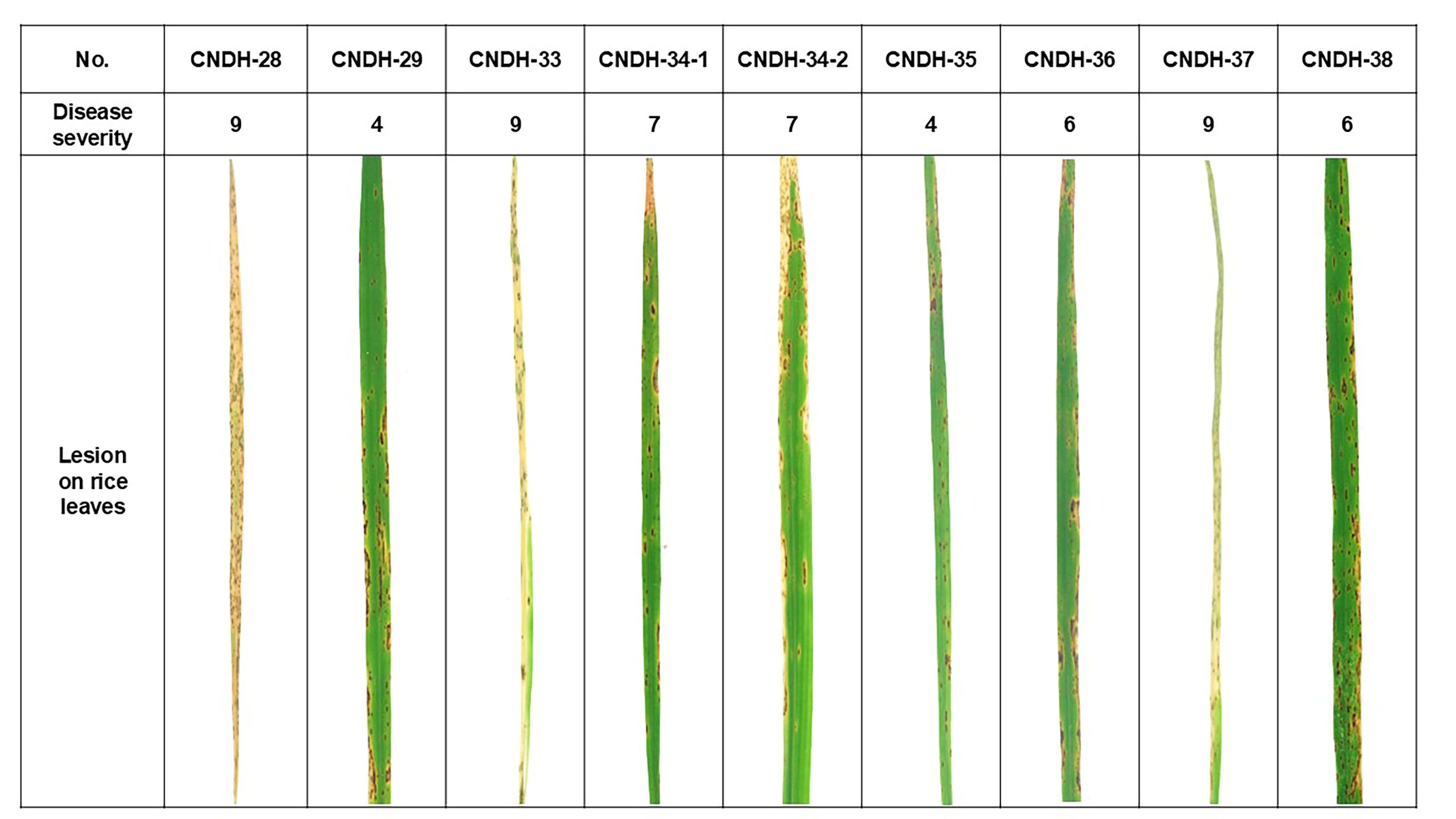
**

**
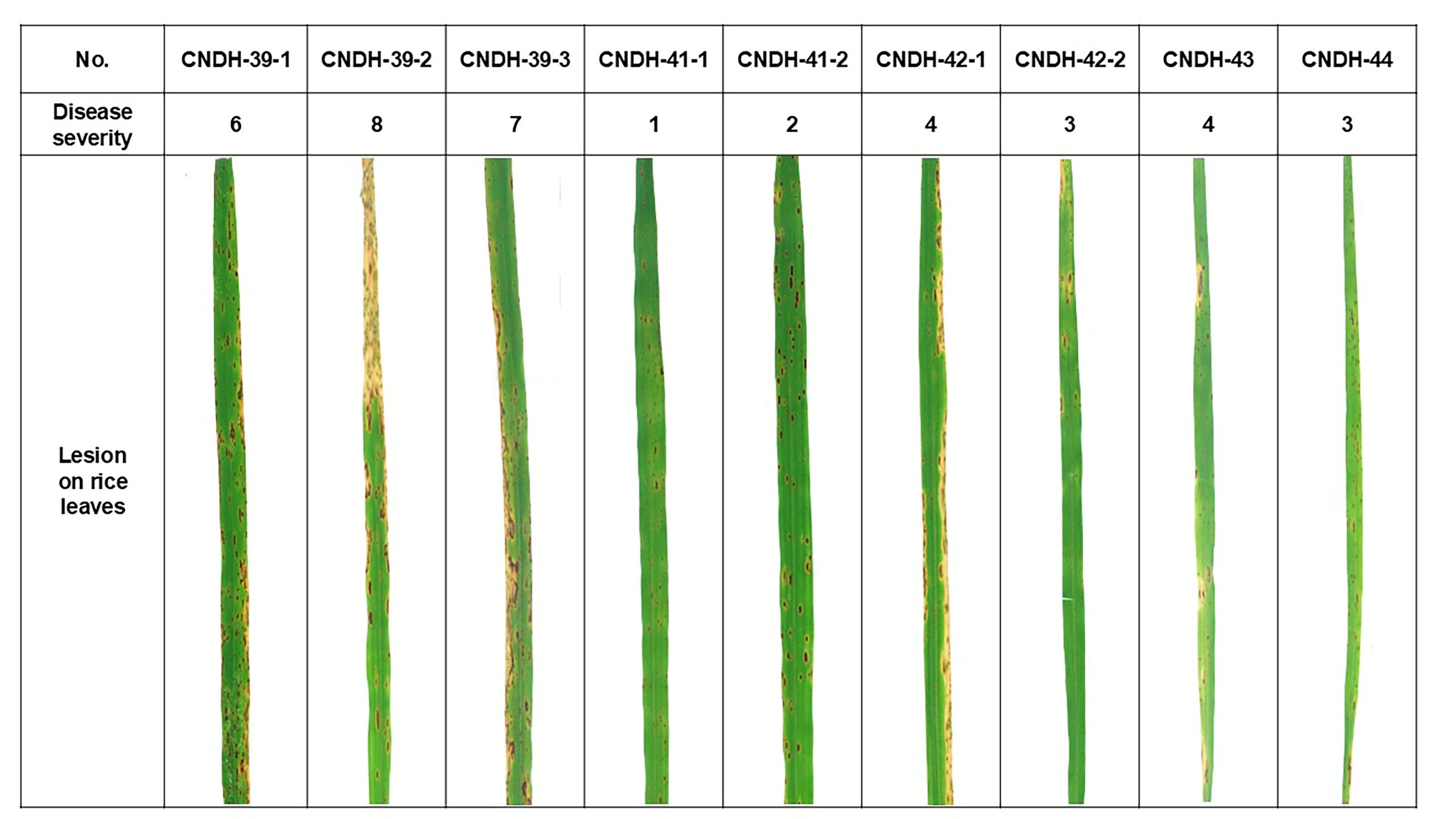
**

**
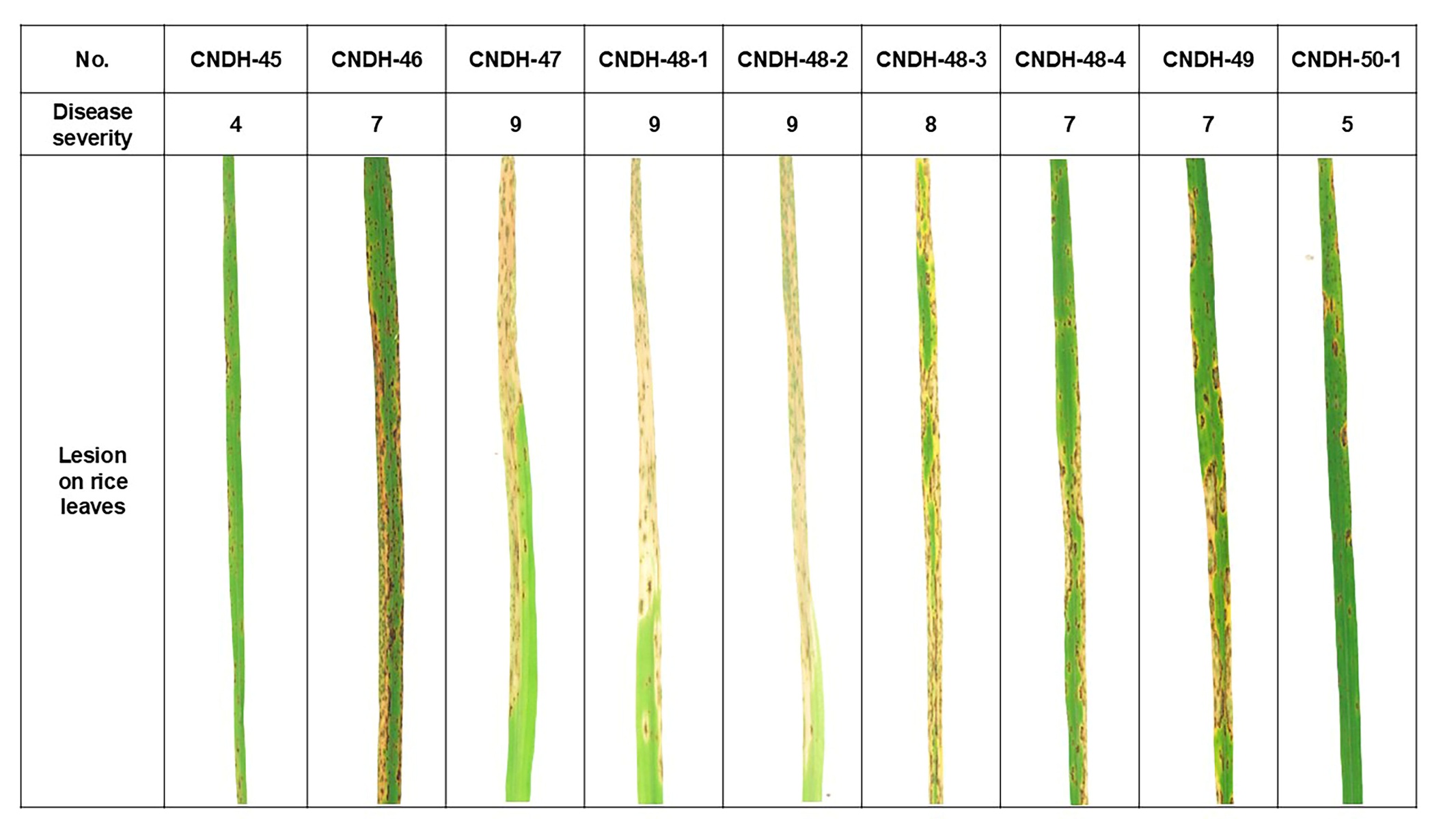
**

**
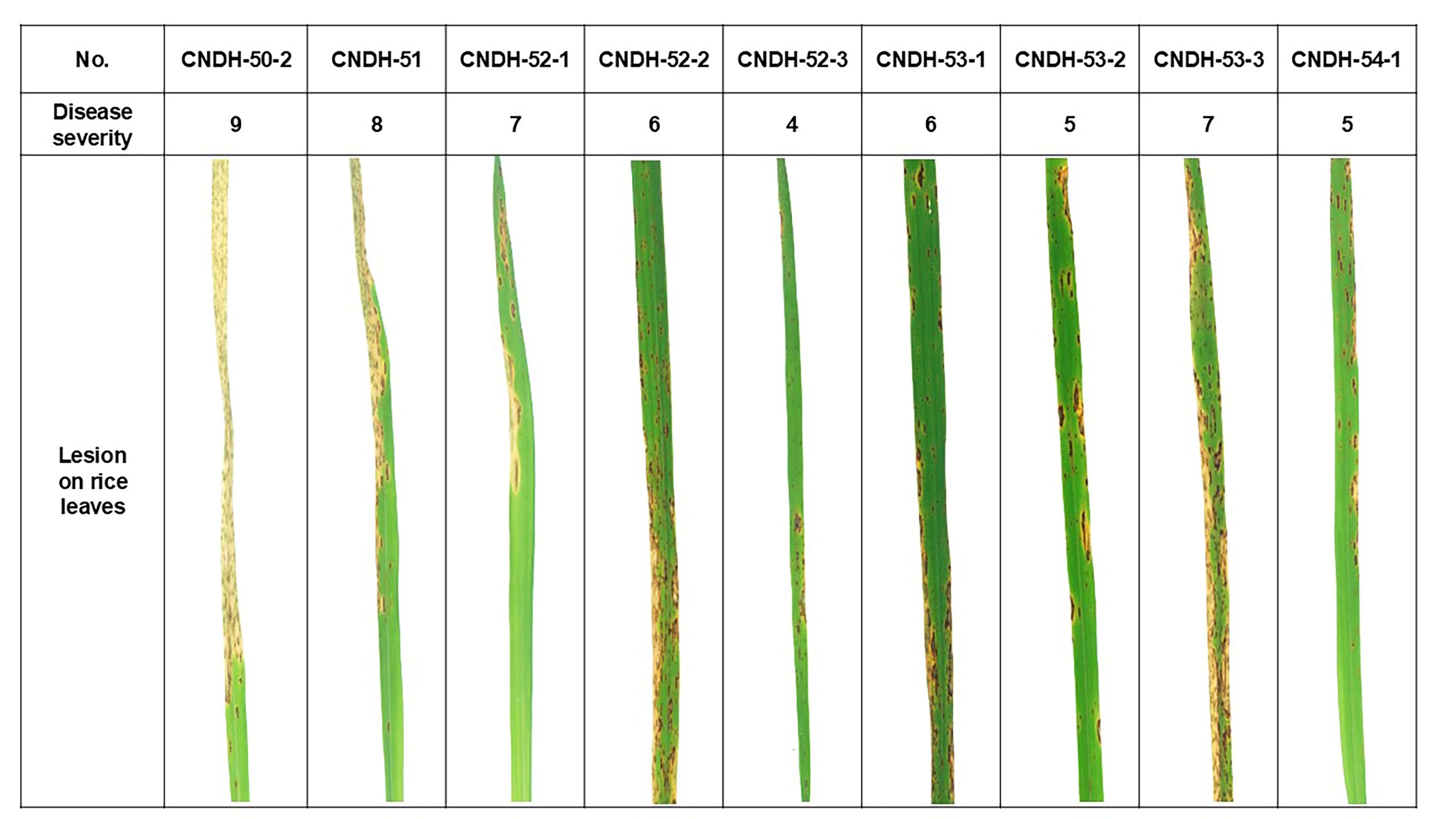
**

**
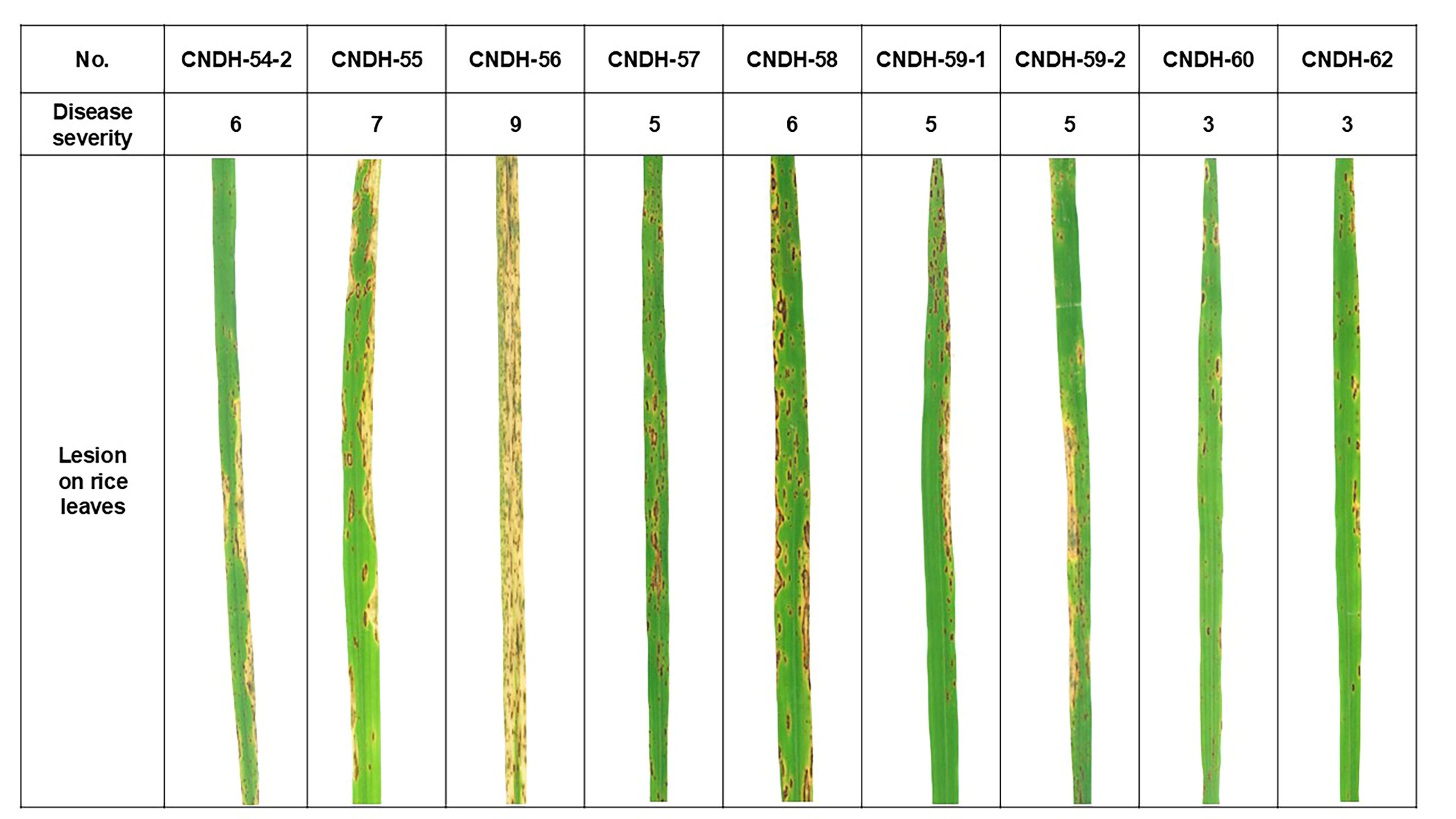
**

**
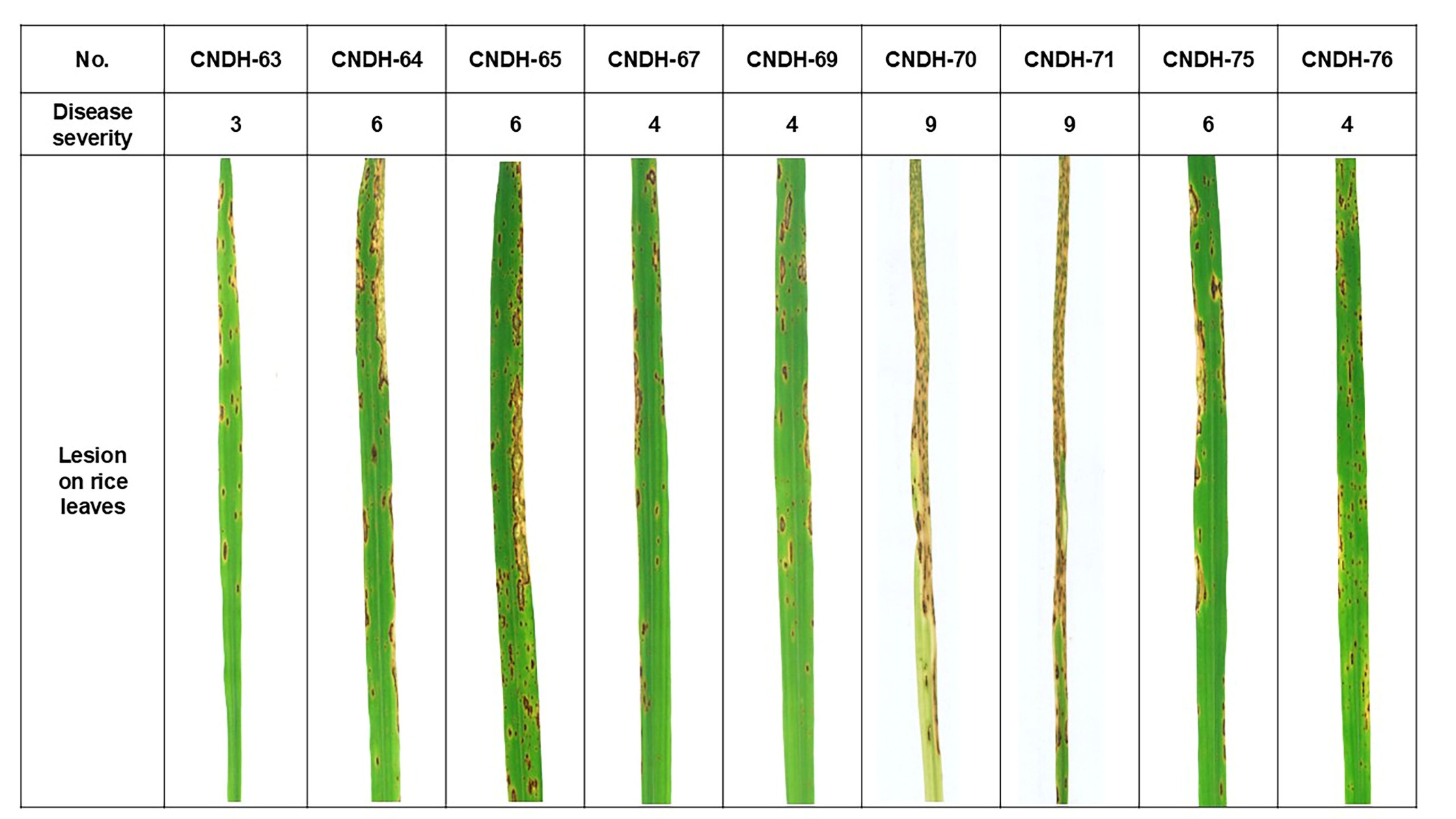
**

**
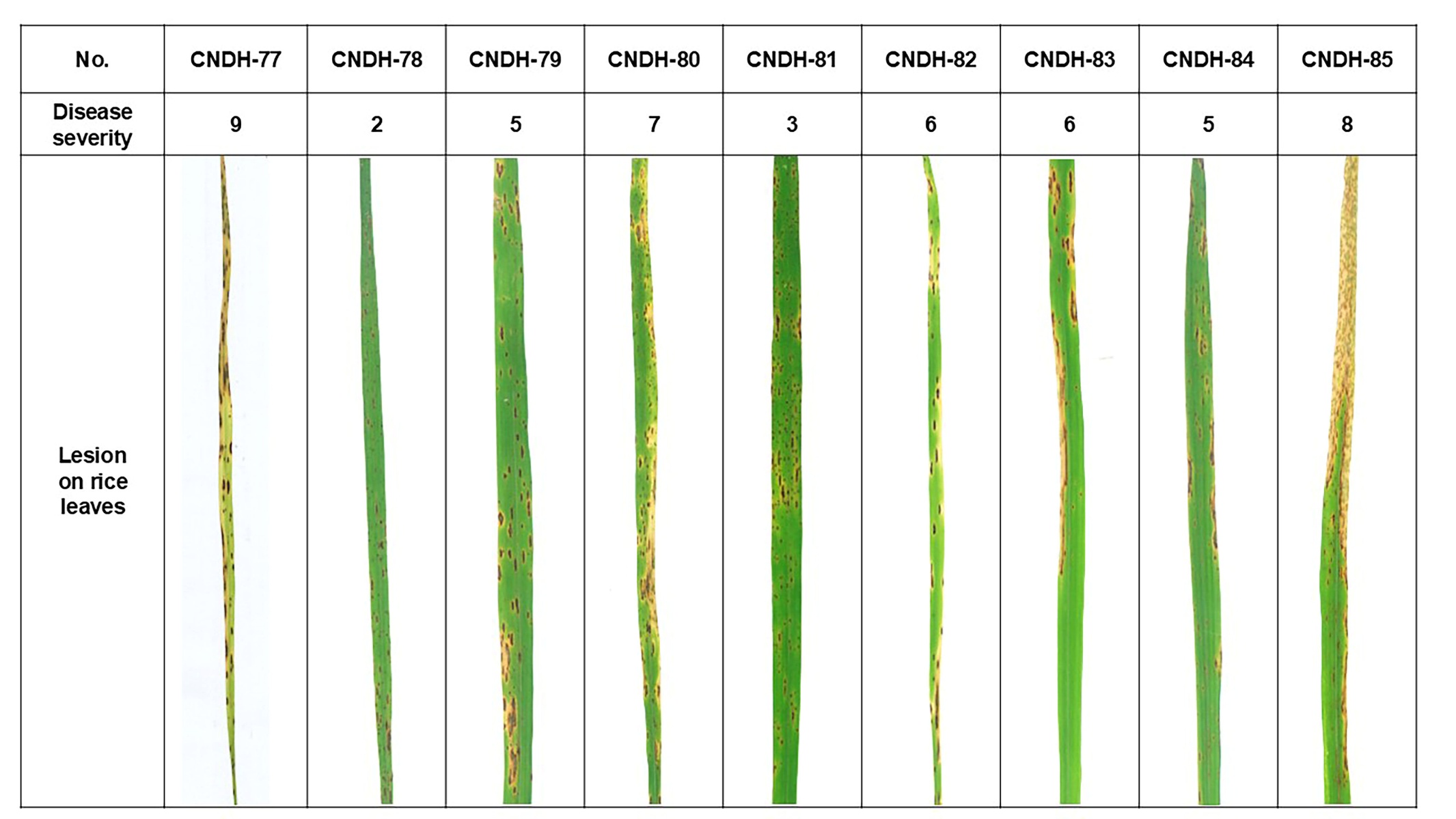
**

**
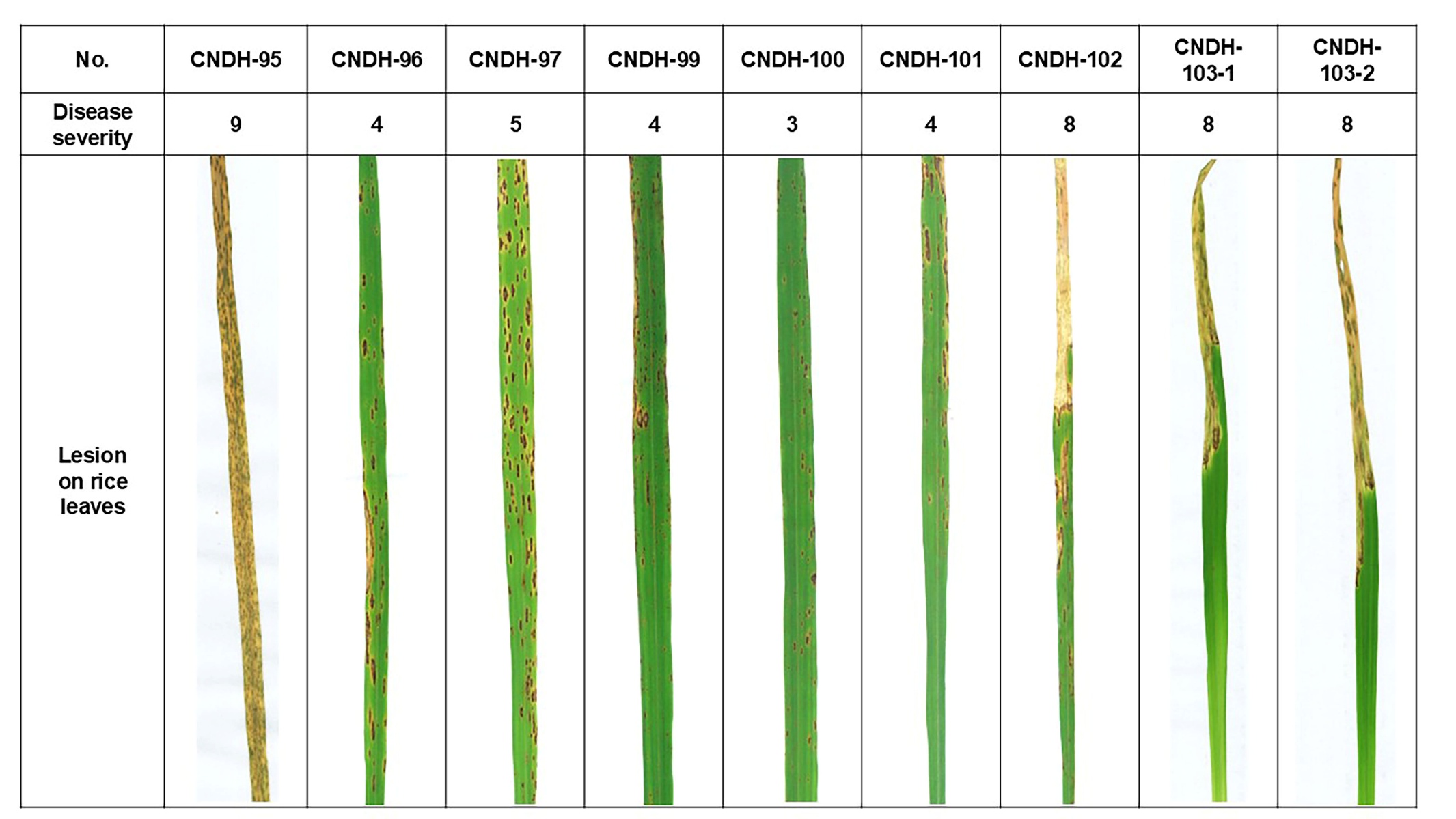

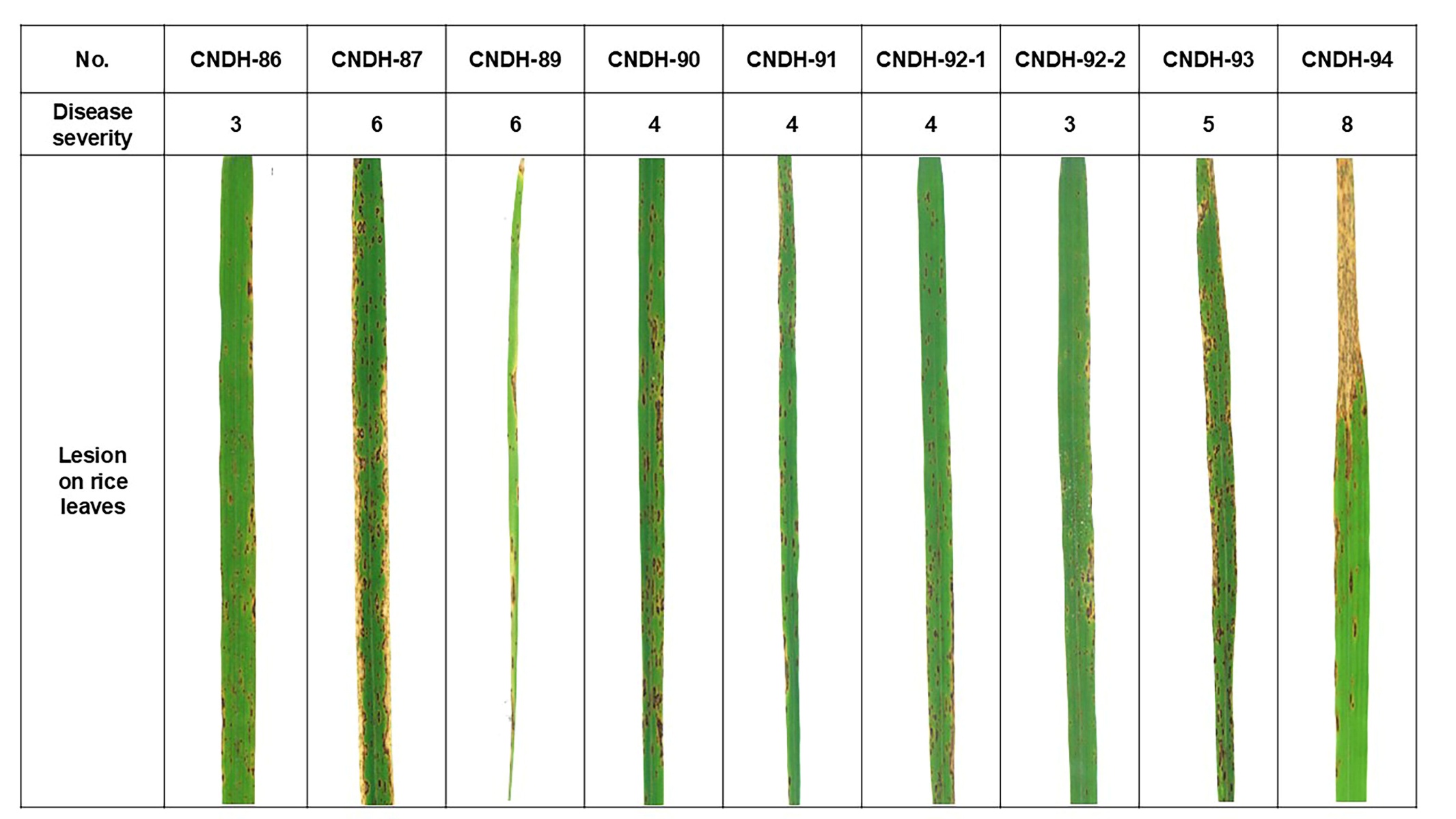
**

**
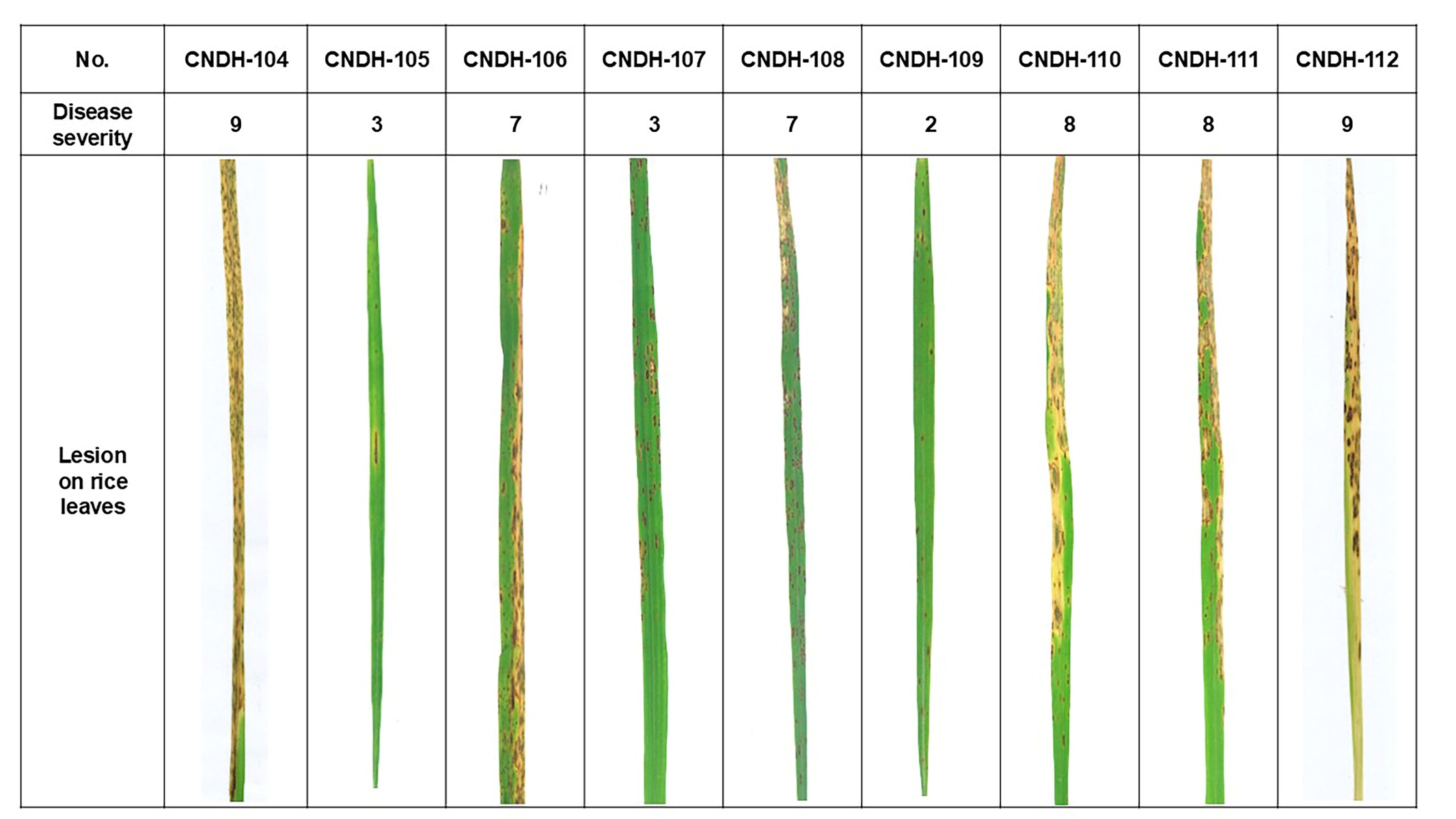

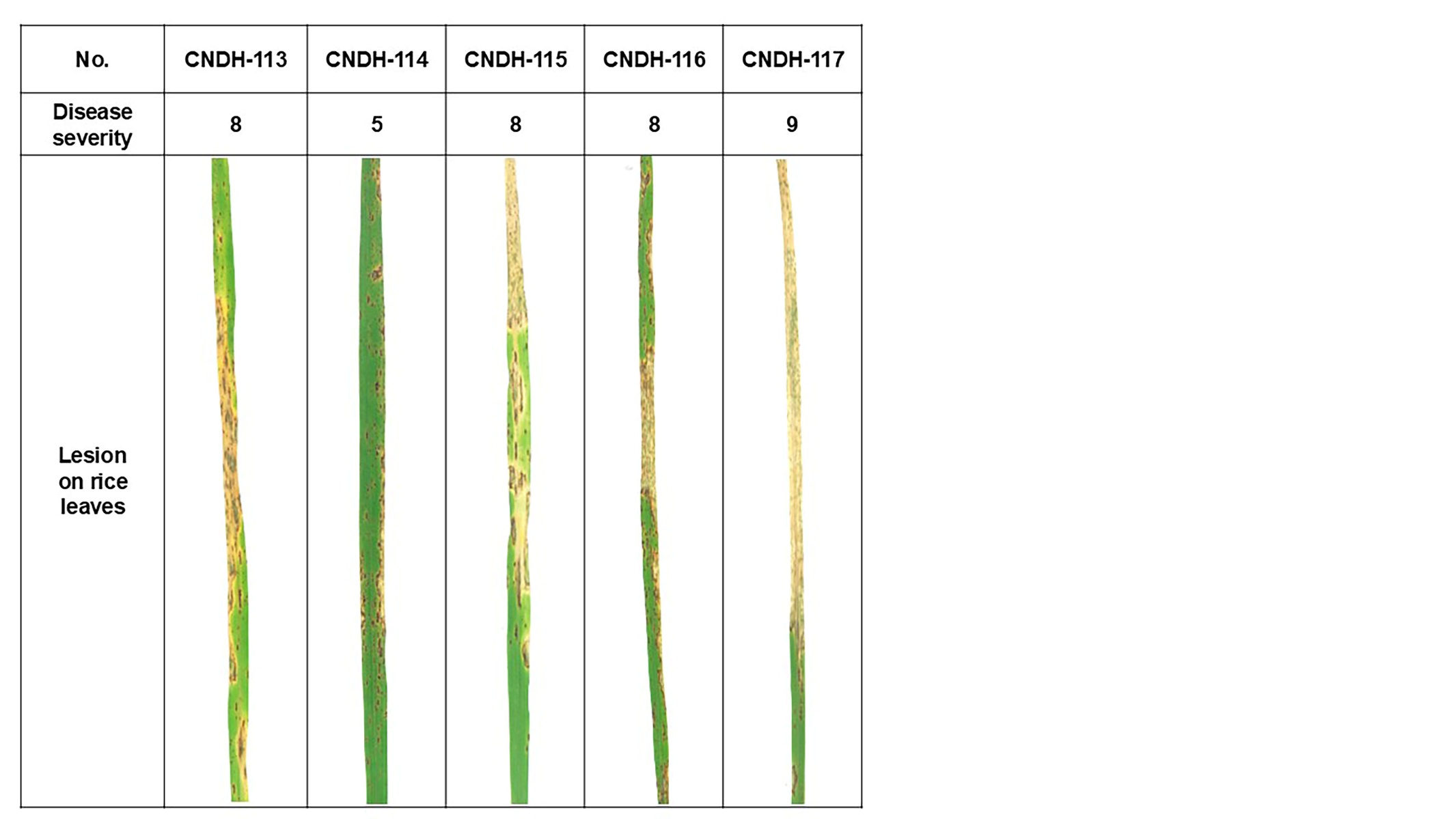
**

**Supplementary Table S2.** QTL related to the rice brown spot disease resistance of the CNDH population.

| **Characteristics** | **Chromosome** | **Locus** | **Marker Interval^a^** | **LOD** | | **Add. Effect^b^** | | **R^2c^** | | **Increasing effects^d^** | |
| --- | --- | --- | --- | --- | --- | --- | --- | --- | --- | --- | --- |
| Rice brown spot disease resistance | 3 | *qRBS3* | RM15749-RM15689 | 2.68 | 0.75 | | 0.21 | | Cheongcheong | |  |

^a^ Marker Interval are those within the significance threshold on each border of the QTL range.

^b^ Additive effect.

^c^ Phenotypic variation explains each QTL.

^d^ Increase effect is the source of the allele causing an increase in the measured traits.

**Supplementary Table S3.** Candidate genes associated with rice brown spot resistance located within the QTL interval flanked by markers RM15749 and RM15689 on chromosome 3.

| **Locus ID** | **Gene Description** |
| --- | --- |
| Os03g0685100 | tRNA/rRNA methyltransferase, SpoU domain containing protein. |
| Os03g0685300 | Glutamine amidotransferase class-I domain containing protein. |
| Os03g0685500 | CHCH domain containing protein. |
| Os03g0685600 | WD40-like domain containing protein. |
| Os03g0685700 | Protein prenyltransferase domain containing protein. |
| Os03g0685800 | Hypothetical protein. |
| Os03g0685900 | Conserved hypothetical protein. |
| Os03g0686100 | Hypothetical protein. |
| Os03g0686300 | Dilute domain containing protein. |
| Os03g0686500 | Conserved hypothetical protein. |
| Os03g0686700 | Conserved hypothetical protein. |
| Os03g0686900 | Glycoside hydrolase, family 13, N-terminal domain containing protein. |
| Os03g0687000 | Conserved hypothetical protein. |
| Os03g0687200 | Conserved hypothetical protein. |
| Os03g0687400 | Ribosome-inactivating protein family protein. |
| Os03g0687700 | Conserved hypothetical protein. |
| Os03g0687800 | Conserved hypothetical protein. |
| Os03g0687900 | Hypothetical protein. |
| Os03g0688000 | Ribosome-inactivating protein family protein. |
| Os03g0688200 | Conserved hypothetical protein. |
| Os03g0688300 | Similar to Calcium-dependent protein kinase. |
| Os03g0689100 | Histidine acid phosphatase family protein. |
| Os03g0689300 | Plasma membrane H+ ATPase (EC 3.6.3.6) (H-ATPase). |
| Os03g0689400 | Disease resistance protein family protein. |
| Os03g0689900 | Tetratricopeptide region domain containing protein. |
| Os03g0690000 | Conserved hypothetical protein. |
| Os03g0690500 | 2OG-Fe(II) oxygenase domain containing protein. |
| Os03g0690600 | Similar to SCARECROW-like protein. |
| Os03g0691400 | Phenylacetic acid degradation-related protein domain containing protein. |
| Os03g0691500 | Conserved hypothetical protein. |
| Os03g0691800 | Similar to HIPL1 protein precursor. |
| Os03g0692000 | Glycosyl transferase, family 14 protein. |
| Os03g0692400 | Protein of unknown function DUF966 family protein. |
| Os03g0692500 | Galectin, galactose-binding lectin family protein. |
| Os03g0692700 | Similar to Pherophorin-S precursor. |
| Os03g0693000 | Protein of unknown function DUF1336 domain containing protein. |
| Os03g0693400 | Protein of unknown function DUF538 family protein. |
| Os03g0693600 | Similar to Indole-3-acetate beta-glucosyltransferase (EC 2.4.1.121) (IAA-Glu synthetase) ((Uridine 5'-diphosphate-glucose:indol-3-ylacetyl)-beta-D- glucosyl transferase). |
| Os03g0693700 | Similar to Oxalate oxidase 1 (EC 1.2.3.4) (Germin). |
| Os03g0693800 | Similar to Oxalate oxidase 1 (EC 1.2.3.4) (Germin). |
| Os03g0693900 | Similar to Oxalate oxidase 1 (EC 1.2.3.4) (Germin). |
| Os03g0694000 | Similar to Oxalate oxidase 1 (EC 1.2.3.4) (Germin). |
| Os03g0694500 | Similar to Permease 1. |
| Os03g0694700 | Conserved hypothetical protein. |
| Os03g0694800 | Ribosomal protein L18P/L5E family protein. |
| Os03g0694900 | Similar to Valyl-tRNA synthetase (EC 6.1.1.9) (Valine--tRNA ligase) (ValRS). |
| Os03g0695400 | Ankyrin repeat containing protein. |
| Os03g0695500 | Protein of unknown function DUF1517 family protein. |
| Os03g0695600 | Proteasome subunit beta type 2 (EC 3.4.25.1) (20S proteasome alpha subunit D) (20S proteasome subunit beta-4). |
| Os03g0695700 | Similar to Chloride channel protein CLC-d (AtCLC-d). |
| Os03g0696000 | Harpin-induced 1 domain containing protein. |
| Os03g0696300 | CCAAT-binding transcription factor, subunit B family protein. |
| Os03g0697200 | Similar to Lysine decarboxylase-like protein. |
| Os03g0698200 | Conserved hypothetical protein. |
| Os03g0698400 | Non-protein coding transcript, unclassifiable transcript. |
| Os03g0698500 | Similar to Yippee-like protein 3. |
| Os03g0698800 | Zinc finger, CCCH-type domain containing protein. |
| Os03g0698900 | Alkaline phytoceramidase family protein. |
| Os03g0699000 | Oleosin 18 kDa (OSE721). |
| Os03g0699100 | WD40-like domain containing protein. |
| Os03g0699200 | Pescadillo, N-terminal domain containing protein. |
| Os03g0699300 | Similar to Adenylosuccinate synthetase, chloroplast precursor (EC 6.3.4.4) (IMP-- aspartate ligase) (AdSS) (AMPSase). |
| Os03g0699400 | Silencing group B protein. |
| Os03g0699600 | Cyclin-like F-box domain containing protein. |
| Os03g0699700 | 9-lipoxigenase. |
| Os03g0699800 | Conserved hypothetical protein. |
| Os03g0700200 | Conserved hypothetical protein. |
| Os03g0700400 | Similar to Lipoxygenase 1 (EC 1.13.11.12). |
| Os03g0700600 | Conserved hypothetical protein. |

**Supplementary Table S4.** List of primers used in this study.

| **No.** | **Locus Name** |  | **Sequence** |
| --- | --- | --- | --- |
| 1 | *Os03g0685100* | F | GCTTCTCTTGGACGGCATAG |
|  | *LOC_Os03g48050* | R | CGAGCCACTCATCAAGAACA |
| 2 | *Os03g0685300* | F | TTGGCGGTAATCAACTCCTC |
|  | *LOC_Os03g48060* | R | ACAAACGACCGGATTCAAAC |
| 3 | *Os03g0685500* | F | AACGGAGGTGGCTCTATCCT |
|  | *LOC_Os03g48080* | R | ACCAGTACCAAAAGCCATGC |
| 4 | *Os03g0686300* | F | ATCGCTTTGGTGTTCTTGCT |
|  | *LOC_Os03g48140* | R | AGTTCTCCAGCCCCATTTTT |
| 5 | *Os03g0686900* | F | AATGGGTCAGAGTGGAGCAC |
|  | *LOC_Os03g48170* | R | GGGATGCAGTACGGAAGAAA |
| 6 | *Os03g0687400* | F | CTGCCGTACATCGAGCACT |
|  | *LOC_Os03g48200* | R | TAATGTTGTTCCCGCCAAGT |
| 7 | *Os03g0688000* | F | ATCCTCTGCAACTCCCAGTC |
|  | *LOC_Os03g48230* | R | CTGCCATCGCTTTAATCACA |
| 8 | *Os03g0689100* | F | AAGGCTCTGGAATGCTTGAA |
|  | *LOC_Os03g48300* | R | GACATTGGGCAAGAGCATTT |
| 9 | *Os03g0689300* | F | GGCACATGCACATAGGACAC |
|  | *LOC_Os03g48310* | R | GGAACATGGCAAAAGCAACT |
| 10 | *Os03g0689400* | F | ACCATTGTCCGAATGTGGAT |
|  | *LOC_Os03g48320* | R | TGGTCTGTTTCTGCATCTCG |
| 11 | *Os03g0689900* | F | GGTCCAGATTTCGATGCACT |
|  | *LOC_Os03g48380* | R | GCTCTGCCATGCAATGAGTA |
| 12 | *Os03g0690500* | F | GCTTCTTCCAGGTGGTCAAC |
|  | *LOC_Os03g48430* | R | CGAACAGGTCGAAGTTGGAG |
| 13 | *Os03g0691400*  *LOC_Os03g48480* | F | GCTCAGCATCAACCACTTCC |
|  |  | R | CCTGCATTTTTCACGCTCTC |
| 14 | *Os03g0692000*  *LOC_Os03g48560* | F | ACTACCCCCTCGTCACACAG |
|  |  | R | TCGGGAATCCAGAACAATTC |
| 15 | *Os03g0692400*  *LOC_Os03g48600* | F | ATCACCTGTGGTTCGCTTTC |
|  |  | R | GCGGGTACATCTCTTTGCAT |
| 16 | *Os03g0692500*  *LOC_Os03g48610* | F | CAATGGGGTGGAAACTATGG |
|  |  | R | CCTCCCATCGACTTGAACAT |
| 17 | *Os03g0693000*  *LOC_Os03g48660* | F | CATTCATCGGAGCAGCACTA |
|  |  | R | CGTCCAGTATCCTTCCTCCA |
| 18 | *Os03g0693400*  *LOC_Os03g48710* | F | CGCTACGTCTACCTCGACATC |
|  |  | R | GACCTTGCTTCCTGCCTTC |
| 19 | *Os03g0694800*  *LOC_Os03g48840* | F | CCTGCAATCACCAAGTTCCT |
|  |  | R | CAGGAATGCCTCTGAACAGC |
| 20 | *Os03g0695400*  *LOC_Os03g48904* | F | CCCTCCTGAGAGTGCCTATG |
|  |  | R | ATGGCGCTTTCTTAGTTGGA |
| 21 | *Os03g0695500*  *LOC_Os03g48920* | F | ATTGGCATTACTTCGGCATC |
|  |  | R | TGCCTCTTGATGCTGTTCAC |
| 22 | *Os03g0695600*  *LOC_Os03g48930* | F | GACGAGGACAAGGTGATGGT |
|  |  | R | CGAATCATATCCAGCAAGCA |
| 23 | *Os03g0696000*  *LOC_Os03g48950* | F | TCTCCTACATCGACGCCAAC |
|  |  | R | GGGATGCTGAGGAAGAACAA |
| 24 | *Os03g0696300*  *LOC_Os03g48970* | F | CAACATCGCAGATGACTGCT |
|  |  | R | CTGTGCGGACATAGCTTGAG |
| 25 | *Os03g0698400*  *LOC_Os03g49140* | F | ATGAGGCCATGGAAAAGTGT |
|  |  | R | ACAAGATGACCCTTGGCAGT |
| 26 | *Os03g0698800*  *LOC_Os03g49170* | F | CGGCTCCGTACCTCACTTAG |
|  |  | R | TGACGGATTTAGGGACTTGG |
| 27 | *Os03g0698900*  *LOC_Os03g49180* | F | GCCACCTTACAGCACGTTTT |
|  |  | R | AGGAGACAGAGGCCAACGTA |
| 28 | *Os03g0699000*  *LOC_Os03g49190* | F | GTTTCGCTTCGCTTCGTAGT |
|  |  | R | CAAGCACAAACTCCCCAGAT |
| 29 | *Os03g0699200*  *LOC_Os03g49210* | F | GAAGAAGGTGGAAGGGAACC |
|  |  | R | CCAAGTCACGAAGAGCATCA |
| 30 | *Os03g0699400*  *LOC_Os03g49230* | F | AGGTGAATCTTGGCATGGAC |
|  |  | R | GAGCACCCTTCGATAAACCA |
| 31 | *Os03g0699600*  *LOC_Os03g49250* | F | CACCTTTGGCCATTCTCAGT |
|  |  | R | TTCCAGCAAAATTCACACCA |
| 32 | *Os03g0699700*  *LOC_Os03g49260* | F | GCATCCCAGCACTAGAGGAG |
|  |  | R | CTCCAAGCAACCTCGTTCTC |
| 33 | *OsActin* | F | CGTCCTCCTGCTTGTTTCTC |
|  |  | R | TAGGCCGGTTGAAAACTTTG |
